# A framework for the identification and classification of homogeneous socioeconomic areas in the analysis of health care variation

**DOI:** 10.1186/s12942-018-0162-8

**Published:** 2018-12-04

**Authors:** Ludovico Pinzari, Soumya Mazumdar, Federico Girosi

**Affiliations:** 1 Translational Health Research Institute, Western Sydney University, Sydney, NSW Australia; 2 Capital Markets CRC, Sydney, NSW Australia; 3 Population Health Intelligence Group, Healthy People and Places Unit, South Western Sydney Local Health District, New South Wales Health, Sydney, NSW Australia; 4 South Western Sydney Clinical School, University of New South Wales, Sydney, NSW Australia

**Keywords:** Homogeneity, Geographic variation, Socioeconomic, Peer groups, Reporting, Disadvantage, Gini index, Clustering, Census area data, Categorical variables

## Abstract

**Background:**

Detecting the variation of health indicators across similar areas or peer geographies is often useful if the spatial units are socially and economically meaningful, so that there is a degree of homogeneity in each unit. Indices are frequently constructed to generate summaries of socioeconomic status or other measures in geographic small areas. Larger areas may be built to be homogenous using regionalization algorithms. However, there are no explicit guidelines in the literature for the grouping of peer geographies based on measures such as area level socioeconomic indices. Moreover, the use of an index score becomes less meaningful as the size of an area increases. This paper introduces an easy to use statistical framework for the identification and classification of homogeneous areas. We propose the Homogeneity and Location indices to measure the concentration and central value respectively of an areas’ socioeconomic distribution. We also provide a transparent set of criteria that a researcher can follow to establish whether a set of proposed geographies are acceptably homogeneous or need further refining.

**Results:**

We applied our framework to assess the socioeconomic homogeneity of the commonly used SA3 Australian census geography. These results showed that almost 60% of the SA3 census units are likely to be socioeconomically heterogeneous and hence inappropriate for presenting area level socioeconomic disadvantage. We also showed that the Location Index is a more robust descriptive measure of the distribution compared to other measures of central tendency. Finally, the methodology proposed was used to analyse the age-standardized variation of GP attenders in a metropolitan area. The results suggest that very high GP attenders (20+ visits) live in SA3s with the most socioeconomic disadvantage. The findings revealed that households with low income and families with children and jobless parents are the major drivers for discerning disadvantaged communities.

**Conclusion:**

Reporting indicators rates for geographies grouped according to similarity may be useful for the analysis of geographic variation. The use of a framework for the identification of meaningful peer geographies would be beneficial to health planners and policy makers by providing realistic and valid peer group geographies.

**Electronic supplementary material:**

The online version of this article (10.1186/s12942-018-0162-8) contains supplementary material, which is available to authorized users.

## Introduction and background

The selection of an appropriate geographic unit of analysis is a key decision for the analysis and interpretation of geographic variation of health-related indicators [1, 2]. It is commonly agreed that the unwarranted variation is not associated with the spatial effects of the geographic unit [3], and detecting variation across geographic areas is often useful only if the units have similar characteristics. Therefore, it is important to define what it means for two geographic units to be comparable.

Take for example indicators denoting the frequency of GP visits. In 2012–2013 there were 2.9 million (12.5%) Australians who were frequent GP attenders, with more than 12 GP visits per year. These individuals were more likely to be older and live in rural areas with the most socioeconomic disadvantage [4]. In terms of socioeconomic status, very high GP attenders (20+ GP visits) were almost twice as likely as low GP attenders (1–3 GP visits) to have lived in areas with the most socioeconomic disadvantage (29% compared to 16%). These demographic and socioeconomic differences accounts for almost one-fourth of the variation in Medicare spending across regions [4, 5], and are strongly correlated with the risk of hospitalization for ambulatory care sensitive conditions [6, 7].

In order to adjust for these differences, standardization for age and socioeconomic status is routinely undertaken to eliminate legitimate or warranted variations [8]. However, these adjustments may not always be sufficient [9] and the analysis can be hampered by the fact that geographic units of analysis are heterogeneous along other dimensions that are associated with the indicator of interest.

One approach to reduce the heterogeneity of the study area, while preserving the meaningfulness of the units of analysis, is to adopt a census geography which includes spatial units which are socially and economically meaningful so that there is a degree of homogeneity in each unit. The central idea behind this approach is that area effects on health have been observed to be stronger in more homogeneous areas [10, 11]. A high level of homogeneity among people and households within each area on a given area level index results in a strong relationship between that area level index and individual level indices [12, 13]. Thus, epidemiologists and geographers have argued that units with greater social homogeneity would be appropriate for studying the associations between unit characteristics and a given health indicator [14]. This property makes them suitable for interpreting variation across similar units.

Following this approach, it is possible to remove the spatial effect of the factors which might influence health by presenting and reporting the variation of an indicator using geographic units that have similar characteristics, better known as “peer groups” [15, 16]. Here the emphasis is not necessarily on the explanation of the variation, but rather on producing a reliable picture of the variation in health indicators across an area, allowing for the variation in standard confounders such as age, gender and socioeconomic status. Rates are usually already adjusted for age and gender, leaving socioeconomic status to be accounted for by a judicious choice of peer groups. Therefore, reporting indicators by similar socioeconomically graded areas of residence provides a useful way to analyse health care variation.

Due to the large number of variables that could be used to measure socioeconomic status in relation to health, a proxy is often chosen based on data availability. In the Australian context, the primary socioeconomic proxy measure used to report the variation of health indicators by national departments and agencies is the Index of Relative Socioeconomic Disadvantage (IRSD), derived by the Australian Bureau of Statistics (ABS) from population census data [17–19]. The IRSD scores each area by summarizing population attributes, such as low income, low education attainment, high unemployment, and jobs in relatively unskilled occupations. For example, an area could have a low score if there are (among other things) many households with low income, many people with poor qualifications or many people in unskilled occupations. For ease of interpretation, areas can be ranked by their IRSD score and are classified into groups (e.g. quintiles or deciles) based on their rank.

In this context, one of the major challenges in choosing a suitable geographic unit relates to an adequate compromise between having a unit large enough to get stable indicator rates and not blurring meaningful local variation [20], while preserving the homogeneous characteristics of the residential population [21, 22].

Traditionally, government agencies use a variety of geographies to report various outcomes and indicators. For example, the Census tracts in the US and the Lower Layer Super Output Areas (LSOAS) in the UK were designed with the intent of being homogenous along different socioeconomic variables. Census tracts were originally designed as being relatively homogeneous with respect to rent, occupation and education [23], and typically contain between 2500 and 8000 inhabitants [24]. Lower Layer Super Output Areas (LSOAS) were constructed out of Output Areas (OAS) and contain 1000–3000 residents. They were designed to be socially homogeneous in terms of housing tenure and dwelling type [25]. The homogeneity measure used in the creation of the 2001 UK census OAS [26] was based on the intra-area correlation introduced by Tranmer and Steel [27], which is defined for a single continuous or dichotomous variable, and will be discussed in more details later in this section.

In Australia, a common census geography used for reporting and mapping is the ABS generated Statistical Area Level 3 (SA3) [28, 29]. SA3s are designed to provide a regional breakdown of Australia and usually have a population of between 30,000 and 130,000 people. In major cities they represent the area serviced by a major transport and commercial hub. In regional areas they represent the area serviced by regional cities with populations of more than 20,000 people. In outer regional and remote areas, they represent areas which are widely recognized as having a distinct identity and have similar social and economic characteristics.

While widely used, the majority of SA3s across Australia are in major cities, where the population composition is more likely to be heterogeneous within a geographic area. Therefore, these reporting units are often too large or diverse to produce representative summary statistics [30]. Smaller spatial units located within the SA3 may thus be misclassified leading to their being grouped into incompatible peer groups. This is a well-known issue in area-based analysis [31] and commonly referred to as the Modifiable Areal Unit Problem [32]. The heterogeneity of the reporting unit may also lead to misinterpreting the variation of the phenomenon under study and produce misleading conclusions on health care performance analysis.

Therefore, using only an index score at a large geography may oversimplify the reporting of an area’s relative socioeconomic disadvantage, and the comparison of variation across a peer group should be accompanied by an evaluation of the degree to which those geographic units in the peer group are internally homogeneous. For this reason, it is always recommended that the homogeneity of a given geography should be evaluated prior to any analysis [11, 21, 33].

Despite the relevance of this issue few researchers have attempted to assess the homogeneity of a geographic area using a socioeconomic index. An attempt was made by Flowerdew [34] to build a new zone system to be used for the publication of Scottish Neighbourhood Statistics. For data zone construction, social homogeneity was assessed using the Townsend index of deprivation [35] and calculated by subtracting the number of the decile containing the lowest score from the decile containing the highest score. This value, however, is clearly affected by outliers and therefore inappropriate for the classification of highly skewed distribution. Moreover, the author does not provide an operational definition of homogeneous area.

A different approach to the definition of homogeneous socioeconomic areas consists in considering the social and economic variables separately. Following this method, Steel and Tranmer proposed a homogeneity measure for the distribution of a multi-category variable, using data from the UK census [36]. This is a variance-based measure, weighted to account for differences in the population size of units across the geographic area. This definition, however, is not helpful to measure the homogeneity of an *ordinal* measure such as a socioeconomic index: the index scores do not represent an amount of disadvantage and as such there is no meaningful arithmetic relationship between the values.

Another complexity in dealing with homogeneity of socioeconomic indices found in the literature is their abstract quality. It is often not clear, for example, what meaning to give to a “homogeneous area” in terms of the socioeconomic characteristics of that area. For instance, the norm is to consider that if a particular area has, on average, a greater proportion of people with a relevant measure of disadvantage then that area may be considered as disadvantaged. The choice of what proportion is an appropriate cut-off is difficult. For example, “having 30% of residents or households classified as deprived has different implications for a district of 200,000 than for one of 20,000” [37]. Unlike absolute deprivation, which refers to a threshold of minimum necessity, such as low-income cut-off, relative deprivation is a comparative measure. Therefore, direct comparisons of deprivation level are appropriate when the unit of analysis is designed to cover roughly equal-sized populations.

In this general setting, a range of summary measures are used to describe relative deprivation for higher-level geographies [38]. An example is the Extent measure of the Index of Multiple Deprivation [38]. This measure uses a weighted combination of the population that live in the three most deprived deciles of the distribution. This analytical approach is useful to compare disadvantaged areas, but it does not provide an operational definition of homogenous area.

For all these reasons, it is necessary to define homogeneity precisely, in order to determine how it should be operationalised and measured.

One approach to enhance the description of socioeconomic disadvantage or any other indicator, is to use the distributional information of the units’ data within each area. This paper proposes a general framework to identify distributional properties of a set of data suitable for the presentation and reporting of comparable information of geographic regions with peers. More precisely, we develop an Homogeneity Index (HI) and a Location Index (LI) with the purpose of measuring respectively the concentration and central tendency of a probability distribution.

In particular, we look at the population distribution in the SA3 IRSD decile category. Conceptually, the HI’s value of a distribution is a number between 0 and 1 that is defined as the degree to which the population is concentrated among the set of categories for that area. For example, in the case of the IRSD decile, an HI of zero expresses minimal concentration and occurs when the population is equally distributed among all decile categories (i.e. an IRSD decile contains 10% of the population). Conversely, an HI value equal to 1 is attained if the whole population is concentrated in a single decile. In the latter case, there is no variation within the area in that characteristic and the geography is uniquely identified by the central value of the distribution.

The LI of a distribution refers to the category which could be considered representative of the entire population in a unit. For example, in the case of the IRSD deciles, an LI of one represents a very disadvantaged area while an LI of ten indicates an area with the lowest level of disadvantage. We propose to use the combination of LI and HI to identify peer groups of spatial units since the LI (or IRSD alone) is not sufficient for this purpose.

Finally, this approach allows us to facilitate the visualization of multivariate data. It is difficult to visualize a large number of variables on thematic maps. Using our approach, individual features such as the incidence of health-related indicators in a specific geography and the characteristics of that area can be combined into a single dashboard with two indices.

**Fig. 1:**
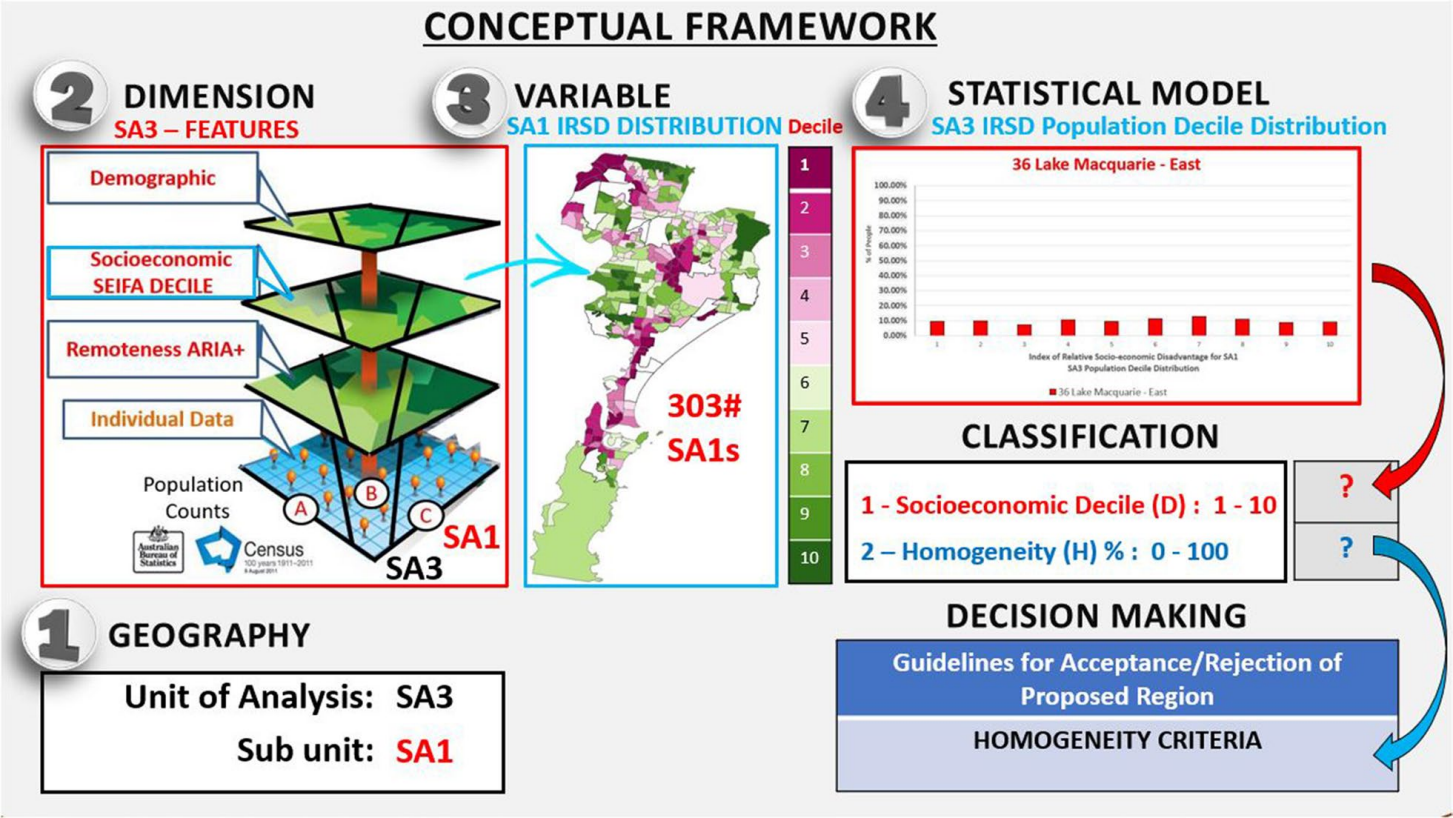
Conceptual framework for the classification of homogeneous areas

In Fig. 1, we illustrate the proposed conceptual framework that could be useful for the evaluation of homogeneous areas in health geographic studies. The first decision is the selection of the larger geographic area (e.g. SA3) and its subunits (e.g. SA1: A smaller ABS geography). Then, the contextual dimension along which one wishes to measure the homogeneity of the geographic area must be defined (e.g. SEIFA, Socio-Economic Indexes for Areas). Third, the selection of the variable used in the model must be specified since measuring the homogeneity among multiple unordered or multiple ordered categories of a variable needs a different set of measurement tools (e.g. IRSD decile). Finally, the selection of the statistical model used to represent the distributional characteristics of the area. We are interested in measuring and operationalising the distribution of a categorical ordinal variable such as the proportion of people in each decile category of the IRSD.

Therefore, we used the above-mentioned framework to answer the following questions:

1. Are the SA3s an appropriate geography for reporting health care variation by socioeconomic status?
2. How homogeneous are these units with regard to the IRSD?
3. Can we use a combination of HI and LI to better find homogenous geographies and therefore identify peer groups better?

This set of analyses uses SA3s to assess the homogeneity of a geographic area. However, the approach can be used to evaluate the socioeconomic homogeneity across any specified geographical boundaries. It is important to notice that the methodology does not require access to fine geographic scale data, and it is easily applied to any distribution of a categorical ordinal variable. Therefore, it requires only the distribution of the attributes for the larger area.

Our approach is founded on the general theory of probability distributions, and our aim is to provide a natural benchmark for a homogeneity measure in terms of what is a “high” (i.e. homogeneous) and “low” (i.e. heterogeneous) concentration of a probability distribution. Currently, there is no accepted benchmark that could be used to assess the homogeneity of a categorical ordinal variable. In this work, we show how the proposed statistical indices can be used to investigate the diversity of a geographic area and determine when the unit of analysis should not be used for reporting health outcomes by socioeconomic status.

The SA3s dataset and the data dictionary have been made available in the Additional file 1 and Additional file 2. The formal definitions and statistical properties of the HI and LI, discussed in the methods section, are illustrated in the Additional file 3. Finally, the Additional file 4 includes the R script to develop these indices.

## Methods

Different disciplines apply different indices to measure the statistical homogeneity or heterogeneity of a given data set. Many indices summarize the data into one number between zero (representing minimum homogeneity) and one (maximum homogeneity). Among these indices, measures of evenness are widely used [39].

Most common in the literature are concentration indices (CI) focusing on the measurement of dispersion among groups defined by nominal categorical variables [40], such as, for example, housing tenure or type, gender, religion and ethnicity [36]. Many social and economic variables, however, are of ordinal nature. Examples include socioeconomic status, self-reported health status [41], level of access to primary care services [42] and remoteness classification [29]. Applying a nominal concentration measure to an ordinal variable is usually not appropriate and could generate misleading results. This issue has been addressed recently by many researchers [43–47], and an axiomatic theory for measuring dispersion of ordinal data has emerged [48]. This accumulating body of research, however, has been criticised for focusing more on measuring polarisation rather than homogeneity [49, 50].

Therefore, there is a need for an alternative specification of the concept of dispersion for ordinal variables. In the rest of the section we will develop an Homogeneity Index for ordinal categorical data based on the idea of combining a nominal concentration measure and a polarized function into a single index, accounting for a number of desired properties. This index is a generalization of the Gini’s coefficient that incorporates the common properties of a polarised measure [48] and meets the basic expectation of a homogeneity measure. The index takes the value zero for the uniform distribution and the maximum value of one for the distribution concentrated in one category. Moreover, considering the structural properties of the index [48, 51], we provide additional criteria for validating and comparing measures of homogeneity when the involved variables assume ordinal nature.

To our knowledge, in the statistical literature no operational suggestions were made regarding the definition of a generalized version of the Gini’s index for ordinal categorical data. The ones that have been proposed [52–54], lack the specificity needed to operationalize the classification criteria. It is, therefore, important for the analyst to provide a set of simple and transparent classification criteria to assess the homogeneity of a geographic unit. Accordingly, we propose a possible framework to map the homogeneity values into the specifications space.

A key issue in the classification process, however, is to identify the basic parameters describing the distribution concentration of the attribute for that geographic area. As we have argued, we want a way of looking at the distributions that reflects both the evenness and the dispersion of an ordinal categorical variable. We will consider first the case of a *nominal* categorical variable.

### Concentration index and true diversity

In this section we draw on the rich literature about diversity indices found in the field of ecology (see Jost [55] and references therein), where diversity is to be understood as the opposite of homogeneity. Given a choice of Concentration Index, in order to really understand what a specific value mean and how it relates to the diversity of the distribution one could compare that value to the CI value of a distribution that can be easily visualized and interpreted. The most easily visualized distribution is one whose categories are equally abundant. It is easy to visualize what a distribution of, say, four equally abundant categories would be like. For instance, if the Gini’s index is chosen as a CI, then all distributions that share the value of the Gini’s index of four equally populated categories must have the same diversity. Finding the diversity of a distribution thus reduces to the problem of finding an *equivalent* distribution (one that has the same value of the index) composed of equally common categories. Therefore, the number of equally abundant categories associated with a particular value of an index gives a simple way to specify the evenness of a distribution. As shown by Jost [55], the key to an intuitive interpretation of the diversity of a distribution lies in the ability to convert a concentration measure into the “effective number of categories”, also known as “true diversity” (*s*). The method of conversion of the Gini’s index to an effective number of categories is presented in Additional file 3: Appendix A3.5.

The importance of this number is that is measured in units of number of categories and hence its scale does not depend on the choice of a specific index. This lets us compare and interpret the diversity of a distribution easily. For example, it is natural to say that a distribution with eight equally common categories is more diverse than a community with four equally common categories.

### Homogeneity Index and true diversity

The notion of true diversity has been developed in the context of nominal categorical variables, in which order does not matter. In the case of ordinal variables the key object of interest must be modified to be distribution of *s* equally abundant categories *clustered on s consecutive bins*. In this case the parameter *s* sets the smallest interval of categories which contains all the data. Consider for example the IRSD decile distribution. As we will explain in the next section, if the HI of four equally populated consecutive deciles (i.e. *s *= 4) is 68.53, then *all* distributions that have a smaller HI’s value are equivalent to a community whose socioeconomic groups are concentrated in *at most* four consecutive deciles. Thus, each distribution can be uniquely allocated to a concentration class according to the value of its homogeneity measure.

Therefore, given the set of the HI’s value which have been assigned to the effective number of categories, we can implement statistical operations on those values using the less than equal (≤) and greater than (>) relationships. This allows us to partition the range of the index values into a number of mutually exclusive and exhaustive equivalence classes in which the natural breaks among classes is determined by the number of categories in the distribution. This partition can make it easier to visualize what a HI’s value means and helps analysts to specify the socioeconomic diversity of a geographic area.

As discussed earlier, a key problem for the quantitative analysis of a socioeconomic index is the definition of what is a “high” and “low” concentration of social disadvantage. This leads to the problem of choosing cut-off points to differentiate the IRSD deciles into broad level of socioeconomic disadvantaged group and determine the maximum effective number of categories for a homogeneous group.

### Homogeneity Index and socioeconomic classification

As indicated in the conceptual framework section, the IRSD includes only variables related to relative disadvantage, and therefore it allows to better distinguish between disadvantaged areas and least disadvantaged areas. This means that the SA1 score distribution in the lower deciles are more spread out than the scores of SA1 in the other deciles [56].

This phenomenon is common in highly skewed socioeconomic distributions and is generally referred in the literature as “clumping” [57]. In this case, a typical rule is to use cut-off points, such as the lowest 40% (deciles 1–4: high disadvantage), the highest 20% (deciles 9–10: low disadvantage) and the rest as the middle group (deciles 5–8: medium disadvantage) [58]. As a result, a reasonable specification for an acceptably homogeneous peer group would be an IRSD decile distribution formed at most four consecutive deciles and a socioeconomic classification based on three levels of disadvantage (low, medium and high). In the next two sections we give the definition and classification criteria for the HI.

### Definition of Homogeneity Index

As indicated in the method section, the HI is a generalization of the Gini’s index for ordinal categorical variables. The two key components underlying the HI are the *Concentration index* (CI) and the *Divergence index* (DI).

The CI is a concentration measure for nominal categorical variables. The properties to be considered necessary for any acceptable CI are: Normalization, Continuity, Symmetry, Strict Schur-Convexity and Value validity [51].

Most of the indices in the evenness literature satisfy the first three properties but only a few meet the requirements of Strict Schur-Convexity and Value validity. Among these indices, the Gini’s index is the most popular and is easily related to the pointwise ordering of the Lorenz curve [59].

Therefore, the CI is defined with reference to the Lorenz curve (LC). The LC plots the cumulative percentage of the population (y-axis) against quantiles or groups of the attribute variable. If the population is uniformly distributed, the LC will be a 45-degree line, known as the line of equality, running from the bottom left-hand corner to the top right-hand corner. In all other cases the curve is convex and lies below the line of equality. Thus, the CI is defined as twice the area between the LC and the line of equality, known as the Lorenz Zonoid [60]. For the computation and definition of CI see Additional file 3: Appendix A1. To determine the conditions for a CI to have Value validity see Additional file 3: Appendix A2.

We note that the definition of the CI can be viewed as a study of the discrepancy between a probability distribution (pdf) and the uniform distribution, but it does not completely reflect the amount of spread that the values of a random variable will take on. For example, different distributions with same LC have different variance. This means that the ordering of the pdf coefficients is not important. Therefore, we introduce an additional element to the computation to the HI, that we call the Divergence Index (DI).

The mathematical definition of the DI is presented in Additional file 3: Appendix 3, but for the purpose of this section it suffices to say that DI is a polarisation measure for ordinal categorical variables. The main properties of the DI are: Normalization, invariance of parallel shifts and simple aversion to median-preserving spreads [48].

The first two properties state that the minimum value (zero) should be taken for the one-point distribution and the “parallel” shift of the entire frequency distribution leaves the index’s value unchanged. The last property states that the transfer of cases from one category into the next, which is closer to the extreme categories (i.e. the first or last category) should result in an increased value of the polarisation measure as it will be closer to the distribution in which half of the population is concentrated at the end points of the distribution. An example of this extremal distribution would be a geographic area where the residents are evenly distributed between the least and most disadvantage socioeconomic quantiles or the youngest and oldest age group. In such a configuration, the variance of any bounded probability distribution is maximum [61]. Therefore, the DI captures the amount of fluctuations about the central location of a distribution as well as the local variation in the data. The DI shares some similarities with the variance. For example, distributions with same mean but different variance have different DI and distributions with different mean or median but same variance must have the same DI value.

Given the definitions of the CI and DI we can finally define the HI of a probability distribution over *n* ordered categories (*P*_*n*_) as follows:

1 $$HI(P_{n} ) = \frac{{CI\left( {P_{n} } \right) + DI\left( {P_{n}^{1} } \right) - DI\left( {P_{n} } \right)}}{{1 + DI\left( {P_{n}^{1} } \right)}}\quad n \ge 3\quad P_{n}^{1} = \left( {\frac{1}{n},\frac{1}{n}, \ldots ,\frac{1}{n}} \right)$$

As anticipated the HI is a number between 0 and 1: it vanishes if and only if the distribution is uniform and it assumes the value of 1 for the singleton distribution. In Additional file 3: Appendix A3.4 we show how the Schur-convexity and Value validity conditions hold for this index.

This allows future comparability if different researchers selected different concentration and polarized functions in the formula.

### Defining homogeneity degree classification

In this section we classify the homogeneity of the IRSD decile distribution, that we denote by *P*_10_, by dividing the HI’s range into four classes of concentration, in which the natural breaks among classes is determined by the number of equally abundant categories in the distribution. The starting point is the observation that it seems to be commonly agreed that a distribution concentrated on at most four consecutive deciles is acceptably homogeneous. We denote any such distribution by *P*_4,10_, and a representative member is for example $P_{4,10} = \left( {\frac{1}{4},\frac{1}{4},\frac{1}{4},\frac{1}{4},0,0,0,0,0,0} \right)$. Therefore, the most homogeneous class of distributions (Class A in Table 1 below) consists of those with an HI higher than the HI of *P*_4,10_, that we denote by *HI*(*P*_4,10_). Applying formula 1, presented in the previous section, and the definitions of CI and DI presented in the Appendices, we obtain that *HI*(*P*_4,10_) = 68.53. For sake of simplicity we shall indicate *HI*(*P*_*s*,10_) as *HI*(*s*) and therefore the most homogeneous class of distributions is defined as follows:

$$Class \; A{:}\, \left\{ {P_{10} {:}\, HI\left( 4 \right) \le HI\left( {P_{10} } \right) \le HI\left( 1 \right),\;\;{\text{where}}\;\;HI\left( 4 \right) = 68.53\;\;{\text{and}}\;\;HI\left( 1 \right) = 100} \right\}.$$

The next homogeneity class, class B in Table 1 below, is obtained in natural way by considering the decile distributions that are not as homogeneous as *P*_4,10_ (*HI *< *HI*(4)) but at least as homogeneous as *P*_5,10_ (*HI *≥ *HI*(5) = 57.62). In formulas:

$$Class\; B{:}\, \left\{ {P_{10} {:}\, HI\left( 5 \right) \le HI\left( {P_{10} } \right) < HI\left( 4 \right),\;\;{\text{where}}\;\;HI\left( 4 \right) = 68.53\;\;{\text{and}}\;\;HI\left( 5 \right) = 57.62} \right\}.$$

We consider these distributions as marginally homogeneous, and their corresponding geographic areas may benefit from some refinement, where some portion of the population is assigned to a different geographic unit. The next homogeneity class, class C in Table 1 below is defined similarly as class B, with the 5th decile substituted with the 6th decile. Its formal definition is as follows:

$$Class\; C{:}\, \left\{ {P_{10} {:}\, HI\left( 6 \right) \le HI\left( {P_{10} } \right) < HI\left( 5 \right),\;\;{\text{where}}\;\;HI\left( 6 \right) = 46.62\;\;{\text{and}}\;\;HI\left( 5 \right) = 57.62} \right\}.$$

Some serious judgment is necessary in order to argue that a population that is uniformly distributed across 6 consecutive deciles is homogeneous, so clearly in these cases homogeneity could be improved by refinement of the geographic areas.

**Table 1:** Homogeneity Index guidelines for acceptance/rejection of proposed region defined by socioeconomic decile distribution

Class	Homogeneity Index (HI) guidelines for acceptance/rejection of proposed region defined by socioeconomic decile distribution *P*_10_		
	Equally populated deciles specification	Range	Decision support system
A	$HI\left( 4 \right) \le HI\left( {P_{10} } \right) \le HI\left( 1 \right)$	$68.53 \le HI\left( {P_{10} } \right) \le 100$	Proposed region is acceptably homogeneous
B	$HI\left( 5 \right) \le HI\left( {P_{10} } \right) < HI\left( 4 \right)$	$57.62 \le HI\left( {P_{10} } \right) < 68.53$	Marginal heterogeneity—reassignment of some units may be beneficial
C	$HI\left( 6 \right) \le HI\left( {P_{10} } \right) < HI\left( 5 \right)$	$46.62 \le HI\left( {P_{10} } \right) < 57.62$	Judgement required whether to accept homogeneous region or to reassign units to other regions to improve homogeneity of current grouping units
D	$HI\left( {10} \right) \le HI\left( {P_{10} } \right) < HI\left( 6 \right)$	$0 \le HI\left( {P_{10} } \right) < 46.62$	Proposed region is heterogeneous—reassignment of some units is needed

*HI*(*s*): HI’s value of s equally populated deciles, s = 1, 4, 5, 6, 10

Finally, the last homogeneity class, class D in Table 1 below, is the one of the distributions that are even less homogeneous than those in class C. We consider this class clearly heterogeneous, and its formal definition is as follows:

$$Class\; D{:}\, \left\{ {P_{10} {:}\, HI\left( {P_{10} } \right) < HI\left( 6 \right), \;\;{\text{where}} \;\;HI\left( 6 \right) = 46.62} \right\}.$$

This classification and the evaluation of the geographic unit are summarized in Table 1.

### Definition of Location Index

Table 1 offers only a picture of the distribution concentration. Describing a distribution statistically also requires determining the location of the data. However, when dealing with ordinal categorical data, the standard measures of centre location, such as the mean or mode, are not appropriate for skewed distributions and often they do not give a meaningful value [41, 62]. For example, homogeneous SA3s are mostly long tail skewed distributions.

As a result, we propose the use of a Location Index (LI) as a new measure of central tendency that is less sensitive to long tailed skewed distributions and outliers. This index identifies the position of the bin in the distribution where the values are mostly concentrated, i.e. the bin such the values of the distribution in its surrounding bins are noticeably higher than the others. The LI is defined precisely in Additional file 3: Appendix B and B1, where we show that is closely related to the median. The difference between the LI and the median lies in its ease of computation and, most important, in the ease of generalization to any number of variables.

The basic idea underlying the LI is to map a nested family of sets around the location of the bin into a single number. If the distribution is univariate the set is a symmetric interval formed by a finite number of categories and the concentration value is given by summing up the likelihood of these regions. In this way, each location has a concentration value and the one with the maximum value corresponds to the point of the LI. Therefore, the maximization of this functional is equivalent to finding the bin with the maximum concentration value. This special property gives the LI a “best guess interpretation”: the LI is the value that is closest to all the other values on a variable when the sign error in guessing does not matter but its magnitude does.

To summarize, we believe that the combination of the LI and the HI’s classification criteria enables users to summarize easily the characteristics of a geography in a single table. This straightforward representation is helpful to evaluate the number of homogeneous areas for each socioeconomic disadvantage group and select a suitable region to identify peer groups geographies as we will show in the next section.

### Example: an application to SA3 geography

In the earlier sections, we claimed that the combination of the LI and HI is important, and we elucidated the theoretical framework behind our claims. In this section, we provide empirical evidence to show that the combination of the LI and HI is a better descriptive statistic than traditional measures. We also show evidence that the LI is a more robust descriptive measure of the distribution location compared to other measures of central tendency. To demonstrate the validity of these indices, we use the Australian census SA3 geography.

Finally, to illustrate the applicability and usefulness of the proposed framework in the analysis of health care variation, we use as an example the variation of GP attendance across a subset of SA3s peer groups.

### SA3s socioeconomic decile classification and Location Index

We begin by attempting to classify SA3s by using exclusively a central tendency measure of the IRSD socioeconomic index. To demonstrate the effective use of the LI on a given data set, we compared this measure to the mean, mode and IRSD score of 331 SA3s, where the IRSD score is created from the population weighted average (PWAVGS) of the SA1 scores within the larger areas [63].

**Fig. 2:**
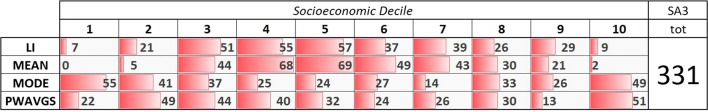
SA3 Location Index (LI) and central measures classification of the Index of Relative Socioeconomic Disadvantage decile distribution

Figure 2 shows the number of SA3s in each socioeconomic group decile for all the statistical measures. The mean tends to cluster the SA3s toward the middle, implying that there is a high risk of misclassification for the lowest and highest socioeconomic categories. The opposite phenomenon is observed with the mode, that is largely concentrated at the end points of the socioeconomic scale. Similarly, the PWAVGS IRSD score is biased by the skewed distributions of scores in the last and first categories. The LI, on the other hand, shows a more symmetric concentration of SA3s in the middle categories with a reasonable number of units in the first and last deciles.

The analysis above shows clearly that the choice of a measure of central tendency can significantly affect the classification of a given geography and the consequent identification of peer groups. In addition, using central tendency alone gives no indication of the diversity of socioeconomic conditions, and SA3s with same value of central tendency may be quite different, as we shown in the next example.

### SA3s comparison in presence of heterogeneity

**Fig. 3:**
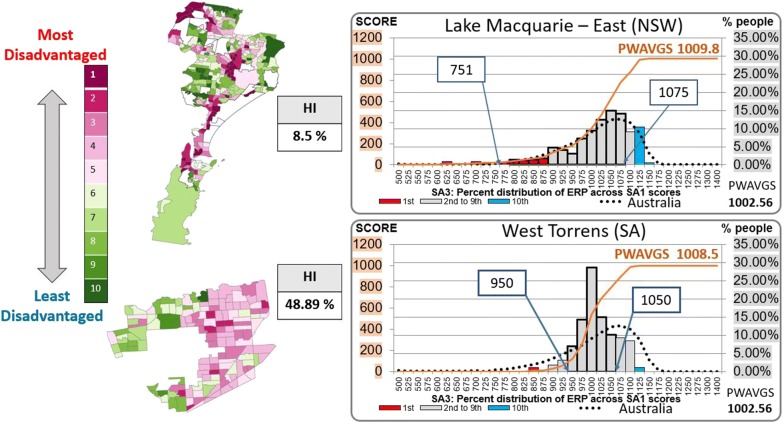
SA3 Index of Relative Socioeconomic Disadvantage distribution comparison—Lake Macquarie-East (#303 SA1s) and West Torrens (#143 SA1s)

In this example we consider the distribution of SA1-level IRSD score within SA3s and use their population weighted average (PWAVGS) score as measure of central tendency. Consider the SA3 of Lake Macquarie-East, located in the Hunter Region of New South Wales. This SA3 received an overall score of 1009.8(*decile*5) on the IRSD. The top chart in Fig. 3 compares the distribution of SA1 scores within this SA3 with that of Australia, depicted with the dotted black curve and overall score of 1002.6(*decile*5). Notice that horizontal axis represents IRSD intervals of size 25 and not deciles. This chart shows that the SA1 scores distribution is quite similar to the one for the entire Australia, suggesting that the SA3 of Lake Macquarie-East is not a homogeneous area. This qualitative analysis is further confirmed by looking at the population decile distribution of Lake Macquarie-East in Fig. 1, where the histogram in red clearly shows that the IRSD decile distribution is almost uniform.

The diversity of this geographic area is highlighted by the thematic map in Fig. 3, which shows the decile distribution of the IRSD for the SA1s within Lake Macquarie-East. Each SA1 is coloured according to its IRSD decile and the legend shows the decile classification. Overall, the map contains many SA1s of varying socio-economic status and, therefore, it is not meaningful to assign a summary IRSD index score to this geographic area. This conclusion is further confirmed by the fact that the true diversity index *s* is equal to 9.2 (not shown in the figure). This means that the distribution is scattered across almost all the 10 deciles and is close to be uniform. Similarly, the Homogeneity Index assumes a very low value of 8.5%, implying that this SA3 belongs to the class D of Table 1 and is therefore clearly heterogeneous.

Thus, whatever central tendency measure we use for the classification of this geographic area, the location value does not truly represent all the people in the area. As a consequence, it is difficult to draw comparisons between this SA3 and, for instance, a SA3 with similar score. Therefore, the use of any location value should always be accompanied by the HI value to acknowledge the diversity within a geographic area.

The usefulness of the HI information becomes clear when comparing two geographic areas with similar score or location value. For instance, the SA3 of West Torrens located in the Western suburbs of Adelaide (SA), received a score of 1008.5(*decile*5), (see Fig. 3) which is close to the score of 1009.8(*decile*5) calculated for Lake Macquarie-East. Based on the central tendency index of the IRSD score, one would conclude that West Torrens is not dissimilar to Lake Macquarie-East. The bottom chart of Fig. 3 shows that that this is clearly not the case, since in West Torrens there is a greater proportion of its population living in the middle scores. In fact, 75% of the residents live in a SA1 with an IRSD score in the range 950–1050, while for Lake Macquarie-East the corresponding range is significantly broader, and equal to 751–1075. This suggests that West Torrens has a higher degree of homogeneity compared to Lake Macquarie-East. In fact, our analysis shows that the HI of West Torrens is 48.89%, which is much higher than the 8.5% of Lake Macquarie-East and places this SA3 in the group C of Table 1.

The higher homogeneity of West Torrens is shown in the map at the bottom left of Fig. 3, representing the geographic distribution of the IRSD at SA1 level. The figure clearly shows that this SA3 could be split in three much more homogeneous regions, justifying its classification in group C.

This example shows why the use of a central tendency indicator, such as the IRSD score, could potentially lead to misclassification of socioeconomically disadvantaged areas. It suggests that both homogeneity and Location Index should be used concurrently for the purpose of understanding the socioeconomic classification of geographic areas. It is therefore important to understand how the SA3 are distributed along both homogeneity and Location Index of the IRSD distribution. We address this issue in the next section.

### SA3s homogeneity and Location Index classification

In this section the socioeconomic variable of interest is still the IRSD index, and we first focus on the distribution of the HI across all SA3. We computed the HI for all the SA3 in Australia and report the results in Table 2. The table shows that the percentage of heterogeneous SA3s (class D) is almost 60% (59.82) and hence a reassignment of some subunits (i.e. SA1s or SA2s) is needed to improve the homogeneity in the study area. The proportion of SA3s in the two most homogeneous classes (A and B) is only 21%, with 19% of the units that need to be evaluated since they could greatly benefit from some level of reassignment (Class C).

**Table 2:** Homogeneity distribution of the Index of Relative Socioeconomic Disadvantage for the SA3 geography

	Homogeneity Index concentration class				Tot
	A	B	C	D	
SA3s	37	33	63	198	331
% SA3	11.18	9.97	19.03	59.82	100

In Table 3 we extend the analysis to include the Location Index, and show the number of SA3s for a given combination of the LI and HI value as a function of the true diversity *s* index, which is easier to interpret than the value of homogeneity. In this table the grey shaded rows indicate the set of SA3s in the first two classes (*HI*(*P*_10_) ≥ *HI*(5)), and the yellow row indicates all the SA3s in the third class (*HI*(6) ≤ *HI*(*P*_10_) < *HI*(5)). Lastly, the remaining light blue rows show the numbers of heterogeneous SA3s (*HI*(*P*_10_) < *HI*(6)).

**Table 3:**
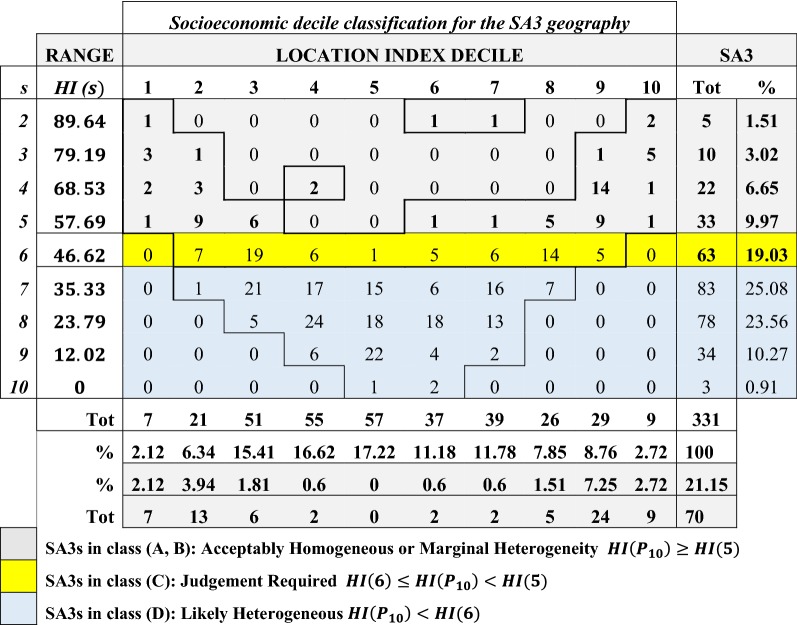
SA3s homogeneity and Location Index decile classification of the Index of Relative Socioeconomic Disadvantage decile distribution

The table shows that the least disadvantaged and most disadvantaged deciles are by far the most homogeneous with respect to the HI value specification threshold (*HI*(5) = 57.69). In particular, most of the SA3 with Location Index in the first or last 2 deciles belong to the homogeneous classes A and B.

This information is further illustrated on the rows below the table, that show the number and percentage of SA3s for each LI value as well as the number and percentage of SA3s in the first two homogeneous classes, indicated in the last two shaded rows.

However, the proportion of SA3s in these categories is less than 5% (i.e. 16 SA3s). A slightly larger number of homogeneous SA3s is in the second and ninth decile, where they account for almost 11% of the SA3s (i.e. 37 SA3s). The middle deciles, on the other hand, have a high proportion of heterogeneous units. Particularly, the least representative decile is the 5th decile and the most heterogeneous unit with an LI equal to 5 is the SA3 of Lake Macquarie-East (*LI *= 5, *HI *= 8.5), indicated in the last row of the 5th column and shown in Fig. 3.

In these examples we emphasized the importance of looking at both measures (LI, HI) in summarizing the socioeconomic disadvantaged of a geography and how to use them in practice. In the next example we show how to these concepts are helpful when analysing the variation of an indicator.

### Reporting Health indicators across SA3s peer groups

To demonstrate the use of the HI and LI, we applied our methodology to the reporting of local variation among GP attenders across the SA3s in the inner and outer metropolitan area of Sydney. Data for this study were sourced from publicly available Medicare Benefits Schedule (MBS) aggregated at the level of SA3, which are administered by the Australian Government Department of Health [64]. These data have two limitations: one is that they are mapped to the areas in which people live, rather than where services were provided, and the other is that they are collected at the level of postal area (POA), that do not have perfect correspondence to the SA3 and therefore require some adjustments.

In this report SA3s have been chosen as an appropriate level of geography to present MBS information as they are large enough in population numbers to ensure confidentiality of MBS statistics, while allowing insights into the variation that exists within a study area. For a technical discussion of the confidentiality requirements the interested reader can refer to the government and state agency report documents [65, 66].

**Fig. 4:**
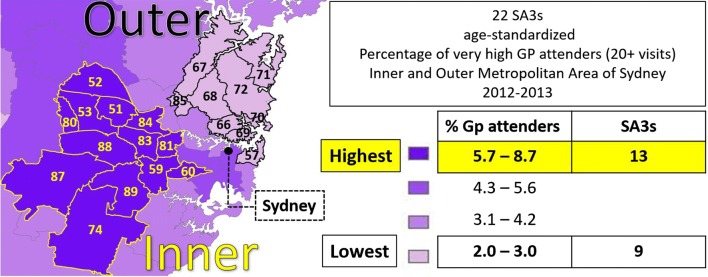
SA3s age-standardized percentage of very high GP attenders (20+ visits) Inner and Outer metropolitan area of Sydney. *Source*: Medicare Benefit Schedule 2012–2013

Figure 4 shows the map of the regional variation of age-standardized percentage of very high GP attenders (20+ visits) across the metropolitan area of Sydney [67]. Results for SA3 were ranked from highest (5.7–8.7%) to lowest (2.0–3.0%) and then split into four groups. The range within each of the four groups is displayed on the right-hand side of the map. The number of SA3s with the highest and lowest percentage of very high GP attenders were 22. Among these, the 13 SA3s with the highest percentage of very high attenders are all in the inner metropolitan area, illustrated with yellow boundaries on the map. The remaining 9, with the lowest percentage of very high GP attenders, are in the outer area, illustrated with dark boundaries on the map.

**Fig. 5:**
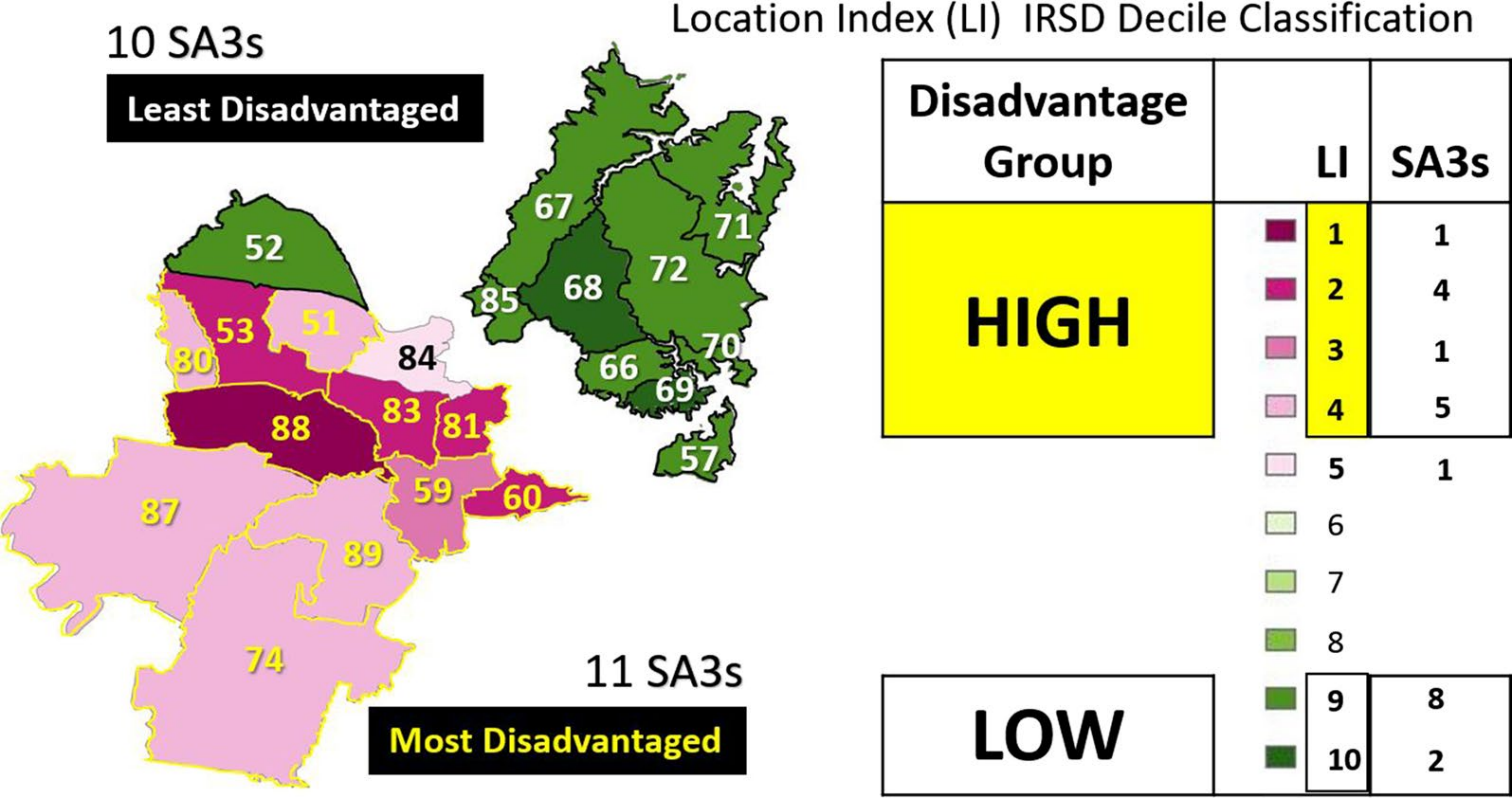
SA3s Location Index (LI) decile classification of the Index of Relative Socioeconomic Disadvantage: inner and outer metropolitan area of Sydney

In order to compare areas on a more equitable basis SA3s were assigned to the high (deciles 1–4) and low (deciles 9–10) IRSD socioeconomic disadvantage group according to the values of their Location Index. The socioeconomic decile classification of these SA3s is illustrated in Fig. 5. The figure shows that, with the exceptions of SA3 52, all the least disadvantaged SA3s are located in the outer area of Sydney and are perfectly aligned with the lowest GP attenders. It follows that the most disadvantaged areas are all in the inner metropolitan area and correspond to the areas with highest proportion of high frequency GP attenders.

**Fig. 6:**
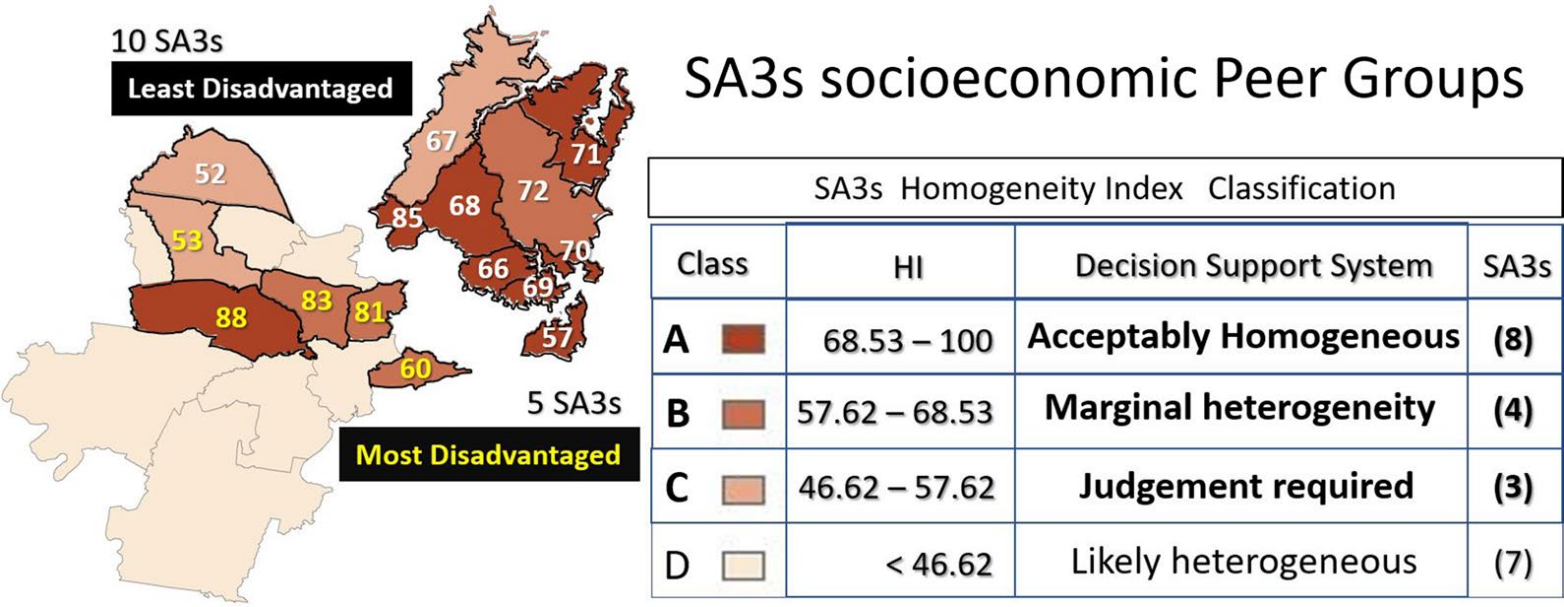
SA3 socioeconomic peer groups of the Index of Relative Socioeconomic Disadvantage for the inner and outer metropolitan area of Sydney

In order to identify comparable SA3s or peer groups we will focus on the first three classes of concentration for the HI (i.e. A, B and C). The map in Fig. 6 shows the number of SA3s in the least and most disadvantaged peer group. All the ten SA3s in the low disadvantage group are in the least disadvantage peer group. On the other hand, five out of eleven SA3s in the high disadvantage group are in the most disadvantage peer group.

**Fig. 7:**
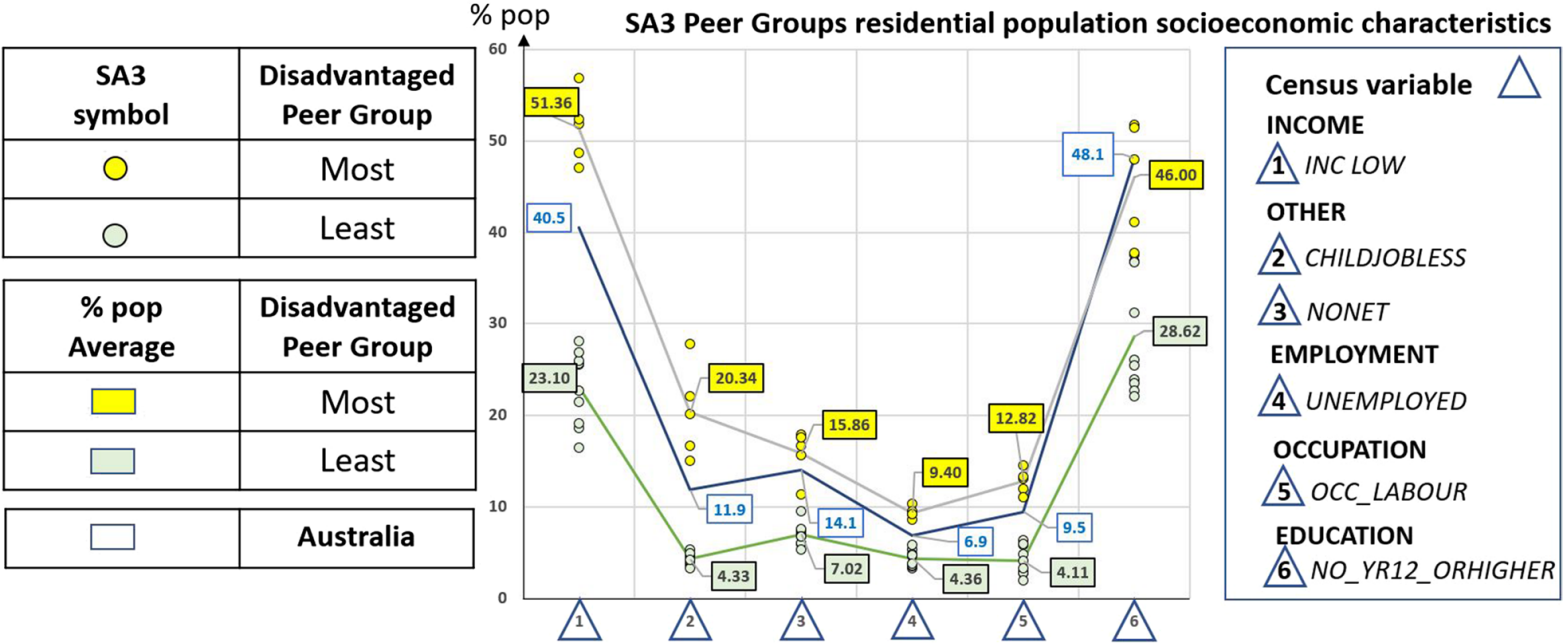
SA3 peer groups residential population census variables of the Index of Relative Socioeconomic Disadvantage for the inner and outer metropolitan area of Sydney

In order to understand the characteristics of these socioeconomic peer groups we drill down and look at the residential population attributes for each of the 15 SA3s. Specifically, we selected a subset of key census variables that contribute to the IRSD SA1 score: income, employment, occupation, education and other indicators of disadvantage, indicated on the right-hand side of the graph in Fig. 7.

An area with most of the disadvantage indicators above the national average will have a low LI. Conversely, spatial units with values below the national average will have a high LI. It follows that indicators which are further away from the national average have a large impact on the LI value.

For example, the graph in Fig. 7 shows that the five most disadvantaged SA3s (i.e. 53, 60, 81, 83, 88) have on average a higher proportion of people with low income ($1 INC LOW:51.36\%$) or families with children under 15 years of age and jobless parents $\left( {2 CHILDJOBLESS:20.34\% } \right)$ compared to the national average $\left( {AUS, INC LOW:40.5\% ;CHILDJOBLESS:11.9\% } \right)$. The ten least disadvantaged SA3s (i.e. 52, 57, 66, 67, 68, 69, 70, 71, 72, 85), on the other hand, have on average a low percentage of $INC LOW \left( {23.10\% } \right)$ and $CHILDJOBLESS \left( {4.13\% } \right)$.

## Discussion

Indices are frequently constructed to generate summary of the socioeconomic status of residential areas. A number of indices have been devised for health research over the years [68–70], including the notably Index of Multiple Deprivation, for identifying the most deprived areas in England [38], and the Townsend’s index designed to explain variation in health in terms of material deprivation [71]. Although these indices are designed primarily to be small-area measures [72, 73], they are also used to describe relative deprivation for higher-level geographies [74, 75].

A major problem facing researchers when constructing indices for larger areas is determining whether the index’s score may be adequate to accurately describe the population living in that area. To facilitate this, a range of summary measures have been designed to help users understand deprivation patterns for higher-level geographies [38]. These methods are helpful to describe the overall intensity of deprivation across the larger area and highlight different aspect of deprivation. However, these analyses focus only to the most deprived small-areas and do not provide a full description of the entire socioeconomic distribution. In addition, they do not offer any guidance for the selection of the geographic area.

An additional problem that arises when working with socioeconomic indices is the classification of skewed distributions. Since the index score is a mean-based measure [38, 63], the computed value is influenced by the extreme scores in the distribution. Consequently, skewed distribution with concentration of scores near the middle can be classified as disadvantaged or least disadvantaged areas. As a result, the averaging effect of the socioeconomic index score chronically under-reports or over-reports disadvantage and can lead to incorrect socioeconomic groups. Similarly, the index score of skewed distributions with concentration around the extreme categories is biased towards the middle.

As a response to these issues, this article presented a framework which is meant to assist in the analysis and reporting of health care variation by identifying homogeneous areas with similar socioeconomic characteristics, better known as peer groups. Therefore, to enable a fairer comparison of individual units with peers, the Homogeneity and Location Index were introduced to measure respectively the concentration and central tendency of a socioeconomic decile distribution. This provides essential information for comparing each group on health indicators of interest.

However, the use of a concentration measure is impractical without knowing how to interpret a given homogeneity value, and especially for ordinal variables there is not much guidance on this subject in the literature. Thus, to better classify and compare the diversity of a geographic area, we have proposed to specify and interpret the HI in terms of the number of equally populated groups in a distribution, also known as “true diversity” [55]. The essence of this approach is that it is a useful and effective method of representing the homogeneity of a community in “picture”, and lets us compare the diversity of a community easily.

Clearly, the specification of the maximum number of equally populated groups that correspond to a homogeneous community depends on the index being analysed. In this work we use the IRSD [56] to represent the socioeconomic conditions of Australian geographic areas and capture aspects of disadvantage. Given the observed patterns in the distribution of the IRSD scores, the lowest 40% of scores was selected to identify the most disadvantaged deciles. As a result, the HI’s threshold for an “acceptably” homogeneous area was determined by a community of four equally populated contiguous deciles.

Other criteria can be chosen for the identification of homogeneous units and there is no definitive or “optimal” HI’s threshold value. However, we believe that greater clarity on this subject is obtained by bringing into the picture the Location Index, that can help users understand when a given area is “highly” homogeneous or heterogeneous. For instance, distributions of six or more equally populated deciles and middle LI values are likely to contain a broader mix of people and households. On the other hand, distributions which have extreme LI values (i.e. very high or very low) and three or less populated deciles are likely to have large proportions of households with similar characteristics.

Therefore, the importance of this representational model lies essentially in its ability to serve as a guide for interpreting dimensionless concentration indices and provide a natural benchmark for these measures in terms of defining what is a “high” and “low” concentration of a probability distribution. This naturally leads to identify geographies where socioeconomic deciles indicated by the LI are meaningful in terms of the HI concentration criteria.

Following this approach, the HI’s range has been partitioned into four classes of concentration, as indicated in Table 1, and an application of these criteria to the Australian SA3 has shown that almost 60% of the census units are likely heterogeneous in terms of IRSD, making comparisons of indicators that correlate with socioeconomic disadvantage difficult to interpret. This result seems highly significant and clearly informs the discussion of which units are appropriate for reporting, suggesting that more work is needed to find ways that allow reporting at lower geographic level while preserving privacy.

The HI’s concentration criteria offer, however, only a partial picture of the distribution classification, and we have argued that one should simultaneously look at the Homogeneity and Location Index. In fact, mapping the Australian SA3s along both these dimensions provide interesting insights. The analysis revealed that the SA3s classified in the least (upper) and most (lower) disadvantaged deciles are by far the most homogeneous units with respect to the concentration criteria. This is expected to some extent, since distributions with Location Index close to the edges have room to expand only on side. What is more surprising, instead, is the lack of homogenous SA3 with Location Index in the centre deciles: SA3s with LI in 5th and 6th deciles are all heterogeneous, with approximately symmetric distributions with long tails. In other words, Australia lacks SA3s in the middle of the socioeconomic disadvantage distribution which are also homogeneous, a fact that seems worth of further investigation.

Overall, it seems that the discriminating power of the LI and HI lies in the lower and upper end of the distribution for identifying the relative disadvantage (lower deciles), and the relative lack of disadvantage (upper deciles) of people in an area. Hence, these indices are particularly suitable for the classification of socioeconomic indices that include variables related to relative disadvantage, such as the IRSD.

To better understand why this might be the case, we used as an example the age-standardized variation of very high GP attenders in the inner and outer metropolitan area of Sydney [67]. To assist comparing areas on a fairer basis, the set of homogeneous SA3s with the greater and lower percentage of GP attenders was divided into two disadvantage categories (i.e. least and most disadvantage).

To identify the deciles cut-off of these socioeconomic groups, we used the 40–40–20 split rule, as in Filmer and Pritchett [58]. We conclude that the interpretation of these deciles is more straightforward for SA3s which have extreme LI values, and that it is usually easy to see why an SA3 which is in the first or last group of deciles has that status.

Overall, this snapshot provided a comprehensive picture of the SA3 socioeconomic characteristics, focusing on people in the lowest and highest socioeconomic groups, where differences are usually large. It can assist in identifying specific populations within urban areas that require health services and resource allocation. It also highlighted that differences can even be seen when restricting comparisons to local areas with similar characteristics and thereby useful to detect the unwarranted variation in health geographic studies.

## Conclusion

Reporting on variation across similar areas aims to assist health care planners in the targeted delivery of health services by identifying areas where to direct efforts to deal with unwarranted variation. Importantly, a key step in studying health outcomes or services utilization is the investigation of spatial patterns of community characteristics by mapping them and by assessing the degree of homogeneity using statistical methods based on individual level information [76].

Many data collections, however, are not released at individual level, and even when they are, they may not include information regarding individual-level socioeconomic position, as in the case of administrative data. This leads to a reliance on area-based information. While area-based measures are relatively easy to collect and utilize at small spatial scales, there is no general definition of homogeneous areas when working with medium or large aggregate population data. Moreover, the use of the index score becomes less and less meaningful as the size of an area increases. Hence, the use of area-based measures to direct support for areas of needs may create a risk for resource misallocations. Therefore, in this paper we introduced an easy to use statistical framework for the identification and classification of homogenous areas.

We applied this framework to assess the socioeconomic homogeneity of SA3, a census geography commonly used in Australia for reporting health indicators. Results from our investigation prompt us to discourage the use of this geography as unit of analysis in the socioeconomic context. The findings also suggest that the proposed framework is a useful tool for strategic planning purpose through its ability to identify areas of disadvantage within broader regions and capture key characteristics of the regions.

Therefore, this tool can be useful in raising discussion on the selection and use of geographic regions (grouping of geographic units) which may be indicative of socioeconomic status. If the region is not acceptably homogeneous, a different definition of the region should be considered. The region could be divided in more subregions or a different assignment of the geographic units to regions could be tried. Another option is the selection of a completely different geographic aggregation unit. Hence, this statistical framework can be naturally embedded in regionalization or clustering methods for building or grouping homogenous regions.

Finally, the combination of the Homogeneity and Location Index constitutes a clear and consistent framework for geographic variation studies. The advantages of such indices include statistical efficiency and a simple presentation of results. They facilitate the visualization of socioeconomic characteristics of geographic areas in a way that can be combined into a dashboard, integrating the homogeneity and central location of the data. This powerful method of illustrating and classifying a geographic area is, therefore, a valuable tool that can act as an interface between the technical and policy disciplines as well as with the decision makers, so they can make scientifically informed decisions.

## Additional files

**Additional file 1.** Dictionary of the SA3’s dataset.

**Additional file 2.** SA3 dataset.

**Additional file 3.** Homogeneity and Location Index definition and properties.

**Additional file 4.** Implementation and documentation of the HI and LI.

## Abbreviations

ABS: Australian Bureau of Statistics
GP: general practitioner
HI: Homogeneity Index
IRSD: Index of Relative Socioeconomic Disadvantage
LI: Location Index
MBS: Medicare Benefit Schedule
PWAVGS: population weighted average score
S: true diversity
SA1: Statistical Area Level 1
SA2: Statistical Area Level 2
SA3: Statistical Area Level 3
SEIFA: Socio-Economic Indexes for Areas
