# Supplementary material for: A framework for the identification and classification of homogeneous socioeconomic areas in the analysis of health care variation
Source: Int J Health Geogr. 2018 Dec 4;17:42. doi: 10.1186/s12942-018-0162-8 (PMC6278138; doi:10.1186/s12942-018-0162-8)
Supplement: Supplementary file 1 — Additional file 1. Dictionary of the SA3’s dataset. [file 12942_2018_162_MOESM1_ESM.docx]

# TABLE DICTIONARY

| Column name | Description |
| --- | --- |
| id | SA3 Sequential identifier |
| SA3_code | ABS code identifier |
| SA3_name | SA3 name |
| state_code | SA3’s state name |
| d1 | Number of people in the first decile of the IRSD |
| d2 | Number of people in the second decile of the IRSD |
| d3 | Number of people in the third decile of the IRSD |
| d4 | Number of people in the fourth decile of the IRSD |
| d5 | Number of people in the fifth decile of the IRSD |
| d6 | Number of people in the sixth decile of the IRSD |
| d7 | Number of people in the seventh decile of the IRSD |
| d8 | Number of people in the eighth decile of the IRSD |
| d9 | Number of people in the ninth decile of the IRSD |
| d10 | Number of people in the tenth decile of the IRSD |
| Hom | The value of the Homogeneity index |
| CI | The value of the Concentration index |
| DI | The value of the Divergence index |
| LI | The value of the Location index |
