# Supplementary material for: A framework for the identification and classification of homogeneous socioeconomic areas in the analysis of health care variation
Source: Int J Health Geogr. 2018 Dec 4;17:42. doi: 10.1186/s12942-018-0162-8 (PMC6278138; doi:10.1186/s12942-018-0162-8)
Supplement: Supplementary file 3 — Additional file 3. Homogeneity and Location Index definition and properties. [file 12942_2018_162_MOESM3_ESM.docx]

# APPENDIX FORMULAS

# **A1: Concentration Index**

Consider a resident population of P individuals and let X= {$X_{1},X_{2},\ldots, X_{i}, \ldots, X_{n}$} be a specific characteristic of the residential population grouped in $n$ categories (e.g. age groups, IRSD quantiles, etc.). Let P(X)= {$p_{X1},p_{X2},\ldots, p_{Xi}, \ldots, p_{Xn}$} be the probability mass function of X (i.e. $p_{Xi}=\frac{f_{i}}{P}, f_{i} absolute frequency : number of people of X_{i}$). The increasing order of the observations is denoted:

$ℿ\left( P\left( X \right) \right)=\{\boldsymbol{p}_{\left( \boldsymbol{1} \right)}\boldsymbol{,}\boldsymbol{p}_{\left( \boldsymbol{2} \right)}\boldsymbol{, \ldots,}\boldsymbol{p}_{\left( \boldsymbol{i} \right)}\boldsymbol{, \ldots,}\boldsymbol{p}_{\left( \boldsymbol{n} \right)}:\boldsymbol{p}_{\left( \boldsymbol{i} \right)}\leq\boldsymbol{p}_{\left( j \right)} \wedge i<j \}$

Then the Lorenz Curve is the piecewise linear connection of points:

$\left( x,y \right)=\left( \frac{k}{n} , \sum_{i=1}^{k} p_{(i)} \right)$, $k=0, \ldots, n \wedge$ $p_{(0)}=0$

in the unit square. Accordingly, the *Lorenz Function* is:

$$\boldsymbol{LF}\left( \boldsymbol{x} \right)=\left\{ \frac{y_{k+1}-y_{k}}{x_{k+1}-x_{k}}\cdot\left( x-x_{k} \right)+y_{k}: y_{k}= \sum_{i=1}^{k} p_{\left( i \right)}\wedge y_{k+1}= y_{k}+p_{\left( k+1 \right)} , x \epsilon\left[ 0\left. 1 \right] \right. \right\}$$

It follows that the area under the Lorenz Curve is:

$$A=\int_{0}^{1} LF\left( x \right)dx (see theorem A1.2)$$

Therefore, the *Lorenz Zonoid* is

$$\boldsymbol{LZ}≝ 2\left[ \int_{0}^{1} x dx-A \right]=1-2A$$

Finally, the CI is given by the following formula:

$$CI=\frac{LZ}{\hat{LZ}} \hat{LZ}= \frac{n-1}{n}$$

The Upper Bound of the Concentration Index is ${(n-1)}/n$ (it is attained when all the observation except one are equal to zero). As a result, the Lorenz Zonoid is divided by $\hat{LZ}$ (See **Theorem A1.1**). It follows that the CI is a function bounded between zero and one.

**Theorem A1.1**: Let P(X) be a probability density function of the random variable

X= {$X_{1},X_{2},\ldots, X_{i}, \ldots, X_{n}$}. Then $\hat{LZ}=max \left\{ CI \right\}=\frac{n-1}{n}$.

*Proof*:

$$\max\left\{ CI \right\}=\max\left\{ LZ \right\}$$

$\max\left\{ LZ \right\}=1-2\cdot min\{A\}$

$\min\left\{ A \right\}=\int_{0}^{1} LF(x,{}_{\arg y_{k}}^{min}{\{A\}) dx}$ →${}_{\arg y_{k}}^{min}{\left\{ A \right\}=\left\{ y_{k}=0 \forall k\neq n \wedge y_{n}=1 \right\}(i.e. singleton)}$

= $\int_{1-\frac{1}{n}}^{1} \left( nx-n+1 \right) dx$ = $n\cdot\left[ \frac{x^{2}}{2} \right]_{1-\frac{1}{n}}^{1}- n\cdot\left[ x \right]_{1-\frac{1}{n}}^{1}+ \left[ x \right]_{1-\frac{1}{n}}^{1}= \frac{1}{2n}$

It follows that:

$\max\left\{ CI \right\}=1-2\cdot\frac{1}{2n}= \frac{n-1}{n}$

***Q.E.D***

**Theorem A1.2**: Let LF(X) be the Lorenz Function of a random variable X= {$X_{1},X_{2},\ldots, X_{i}, \ldots, X_{n}$}. Then, the area under the Lorenz Curve is $A=\sum_{k=0}^{n-1} \frac{1}{n}\cdot\left( \frac{y_{k}+y_{k+1}}{2} \right).$

*Proof*:

Reminding the definition of LF(X):

$$LF\left( x \right)=\left\{ \frac{y_{k+1}-y_{k}}{x_{k+1}-x_{k}}\cdot\left( x-x_{k} \right)+y_{k}: y_{k}= \sum_{i=1}^{k} p_{\left( i \right)}\wedge y_{k+1}= y_{k}+p_{\left( k+1 \right)} , x \epsilon\left[ 0\left. 1 \right] \right. \right\}$$

Considering the following identities:

$$\left\{ \begin{aligned} x_{k}= \frac{k}{n} k=0, \ldots, n \to x_{k+1}= x_{k}+ \frac{1}{n} \\ y_{k+1}= y_{k}+ p_{k+1} \end{aligned} \right.$$

We can compute the Lorenz Curves’ Area:

$$A= \int_{0}^{1} LF\left( x \right)dx= \sum_{k=0}^{n-1} \int_{x_{k}}^{x_{k}+\frac{1}{n}} n\cdot\left( p_{k+1} \right)\cdot\left( x-x_{k} \right)+ y_{k} dx$$

$$=\sum_{k=0}^{n-1} \left\{ n\cdot\left( p_{k+1} \right)\cdot\left[ \frac{x^{2}}{2} \right]_{x_{k}}^{x_{k}+\frac{1}{n}}-n\cdot\left( p_{k+1} \right){\cdot x}_{k}\left[ x \right]_{x_{k}}^{x_{k}+\frac{1}{n}}+ y_{k}\cdot\left[ x \right]_{x_{k}}^{x_{k}+\frac{1}{n}} \right\}$$

$$=\sum_{k=0}^{n-1} \frac{1}{n}\left( \frac{y_{k}+y_{k+1}}{2} \right)$$

***Q.E.D***

# **A2: Value validity CI**

To determine the condition for a CI to have Value validity, Kvålseth recently introduced the lambda distribution **(λ-distribution**) [6]. The term lambda distribution has been chosen for the distribution by Kvålseth because of its L-shape (Greek lambda: **λ**) from. For nominal categorical variable the **λ-distribution** is a single parameter distribution where the parameter **λ** reflects the uniformity or evenness of the distribution and $n$ the number of categories:

$$P_{n}^{\lambda}=\left( \begin{matrix} 1-\lambda+\frac{\lambda}{n}, & \frac{\lambda}{n} \end{matrix},\begin{matrix} \cdots, & \frac{\lambda}{n} \end{matrix} \right)=\lambda\cdot P_{n}^{1}+\left( 1-\lambda\right)\cdot P_{n}^{0} : \lambda\in\left[ \begin{matrix} 0 & 1 \end{matrix} \right] (2)$$

Basically, it is a weighted mean of the one-point distribution $P_{n}^{0}=\left( 1,0,\cdots,0 \right)$, sometimes called singleton or degenerate distribution, and the uniform distribution $P_{n}^{1}=\left( \frac{1}{n},\frac{1}{n}, \cdots,\frac{1}{n} \right)$. Note that a concentration measure for nominal variables is a (permutation) symmetric function and therefore any choice of the one-point distribution could be made. However, we shall consider $1-\lambda+\lambda/n$ to be the first element of $P_{n}^{\lambda}$ and 1 to be the first element of $P_{n}^{0}$. Then, λ is a rectangularity (uniformity) parameter with increasing λ indicating a decreasing approach of $CI(P_{n}^{\lambda})$ from the singleton located in the first category to the rectangular (uniform) distribution $CI(P_{n}^{1})$. Then, a concentration measure has **Value validity** if it regularly decreases from the one-point distribution to the uniform distribution. This means that adding or subtracting an amount δ to or from λ should cause the same absolute change in the value of CI. This is equivalent to define the CI as a function of a single variable λ, that is $CI\left( P_{n}^{\lambda} \right)=CI\left( \lambda\right)=1-\lambda$. It follows that all the CI values lies on the line segment between $P_{n}^{0}$ and $P_{n}^{1}$.

# **A3: Divergence Index**

In the statistical literature, for ordinal types of data, are known lots of indicators to measure the degree of the polarization phenomenon. Typically, many of the widely used measures of distributional variability are defined as a function of a reference point, which in some “sense” could be considered representative for the entire population. This function indicates how much all the values differ from the point that is considered “typical”. Of all measures of variability, the variance is a well-known example that use the mean as a reference point. However, mean-based measures depend to the scale applied to the categories and are highly sensitive to outliers.

Then, is there another way to compare the dispersion of a distribution that does not depend on its location?

To address this challenge, we propose a new measure of polarization, the Divergence Index (DI). The definition of the DI is based on a new representation of probability measures, the Bilateral Cumulative Distribution function (**BCDF**), which derives from a generalization of the Cumulative Distribution Function (CDF). Basically, it is an extended CDF that can be easily obtained by folding its upper part, commonly known as Survival function or complementary CDF. Unlike the CDF, this functional has a finite constant area independently of the probability distribution and, therefore, more convenient for any distribution comparison. For the definition of the **BCDF** see (**A3.1: Bilateral Cumulative Distribution Function**). For the area invariance property see theorem A3.1.2.

Accordingly, the DI is a non-parametric measure that compares the BCDF of two data sets. More specifically, the idea is to compare the distribution shape with that of minimum dispersion, that is the singleton distribution. On this basis, we completely defined the shape of a probability distribution by its BCDF autocorrelation function (**BCDFA**). The main advantage of this representation is that it is invariant to the location of a distribution and therefore is only sensitive to the distribution shape. For example, any singleton distribution will have the same BCDFA curve. Similarly, distribution with same shape but different means and medians can be represented with a unique curve. In this way, to quantify the distance between a pdf and the singleton distribution, it is only a matter of choosing an appropriate metric, which would assign large values to distribution with more dispersed BCDFA than the singleton. For the definition of the **BCDFA** see (**A3.2: Bilateral Cumulative Distribution Function Autocorrelation**).

The selection of a measure to compare probability distribution is not a trivial matter and usually depends on the objectives. In this work we propose the use of the Jensen Shannon Divergence (JSD) [7]. Since the DI is based on the JSD we dub this new measure of polarization **DI-JSD**. For the definition see (**A3.3: DI-JSD**). This measure does not need to be normalized since is a bounded value in the unit interval see **theorem A3.3.1.**

# **A3.1: Bilateral Cumulative Distribution Function**

Let $f\left( X \right)=P(X=X_{i})$ be the probability mass function of the discrete random variable

X = $\left\{ X_{1}, \ldots, X_{i}, \ldots, X_{n} \right\}$ such that $X_{i}$ is a categorical ordinal variable of x (e.g. Age, IRSD score, etc.). It follows that:

$$X_{i}=\left\{ x_{m_{i}}\leq x \leq x_{M_{i}}: f\left( X_{i} \right)=P\left( x_{m_{i}}\leq x \leq x_{M_{i}} \right)=P\left( X_{i} \right)= p_{i} \right\} i \in\{1, \ldots, n\}$$

Let Y = $\{Y_{1}, \ldots, Y_{n} , Y_{n+1} , \ldots, Y_{2n-1}\}$ be a discrete random variable such that:

$$Y_{i}= \left\{ \begin{aligned} X_{i} i \in\left\{ 1, \ldots, n \right\} \\ \\ X \backslash\bigcup_{j=1}^{k} X_{j}=\bigcup_{j=1}^{n} X_{j}\backslash\bigcup_{j=1}^{k} X_{j} i=n+k \wedge k\in\left\{ 1, \ldots, n-1 \right\} \\ \\ \emptyset otherwise \end{aligned} \right.$$

Then the *Bilateral Cumulative Distribution Function* **BCDF** of X is given by the following formula:

$$F\left( Y_{i} \right)\left\{ \begin{aligned} P\left( x \leq x_{M_{i}} \right)=P\left( \bigcup_{j=1}^{i} X_{i} \right)= \sum_{X=X_{1}}^{Y_{i}} f\left( X \right) i \in\{1, \ldots, n\} \\ \\ P\left( x_{m_{k+1}}\leq x \leq x_{M_{n}} \right)=P\left( X_{n} \right)-P\left( \bigcup_{j=1}^{k} X_{j} \right)=1- \sum_{X=X_{1}}^{Y_{k}} f\left( X \right) i \in\{n+1, \ldots, 2n-1\} \\ \\ \\ P\left( x< x_{m_{1}} \vee x> x_{M_{n}} \right)=P\left( \emptyset\right)=0 otherwise \end{aligned} \right.$$

It follows that the recursive definition is:

$$\boldsymbol{F}\left( \boldsymbol{Y}_{\boldsymbol{i}} \right)\boldsymbol{≝}\left\{ \begin{aligned} \boldsymbol{F}\left( \boldsymbol{Y}_{\boldsymbol{i-1}} \right)\boldsymbol{+P}\left( \boldsymbol{X}_{\boldsymbol{i}} \right)\boldsymbol{i \in\{1, \ldots, n\}} \\ \\ \boldsymbol{1-F}\left( \boldsymbol{Y}_{\boldsymbol{k}} \right)\boldsymbol{i\in}\left\{ \boldsymbol{n+1, \ldots, 2}\boldsymbol{n-1} \right\}\boldsymbol{\to k \in\{1, \ldots, n-1\}} \\ \\ \boldsymbol{0 otherwise} \end{aligned} \right.$$

It is worth noting that:$F\left( Y_{i} \right)=CDF\left( X \right)$ for $i \in\left\{ 1, \ldots, n \right\}$ → $\int f\left( X \right)dx=CDF(X)$ and $\int1-CDF\left( X \right)dx=E(X)$ . Basically, the BCDF$=CDF \bigcup CCDF$ Complementary CDF.

Considering, therefore, the recursive definition and the integral relation we can compute the BCDF in terms of **the convolution operator** (see **Theorem A3.1.1**).

**Theorem A3.1.1**: **Convolution operator**

Let $F(Y)$ be the BCDF of the discrete random variable X [see A3.1] and P(X) = $\left\{ p_{1}, .. , p_{i}, \ldots, p_{N} \right\}$ the corresponding probability mass function vector. Let $h\left( X \right)= \left\{ 1 :X=X_{i} \right\}$ be a n-dimensional unitary vector, then $F\left( Y_{i} \right)=p\left[ X \right]*h\left[ X \right]= \sum_{k=-\infty}^{+\infty} p[X_{k}]\cdot h[i-k]$.

*Proof*:

Since $h(i-k)$ is simply the folded sequence $h(-k)$ shifted to the right by $k$ units (if k is positive) or to the left (if k is negative), it follows:

$$F\left( Y_{i} \right)=Y\left[ i \right]= \sum_{k=1}^{N} p\left[ X_{k} \right]\cdot h\left[ i+1-k \right] i \in\{1, \ldots, 2n-1\}$$

$$Y\left[ 1 \right]=p\left[ X_{1} \right]\cdot h\left[ 1 \right]= p_{1} i=1$$

$$Y\left[ 2 \right]=p\left[ X_{1} \right]\cdot h\left[ 2 \right]+p\left[ X_{2} \right]\cdot h\left[ 1 \right]=Y\left[ 1 \right]+ p_{1} i=2$$

$$\ldots$$

$$Y\left[ N \right]=p\left[ X_{n-1} \right]+ p_{N}=Y\left[ N-1 \right]+ p_{N}= 1 i=N$$

$$Y\left[ N+1 \right]= \sum_{k=1}^{N+1} x\left[ k \right]\cdot h\left[ N+2-k \right] i = N+1$$

$$=x\left[ 1 \right]\cdot h\left[ N+1 \right]+x\left[ 2 \right]\cdot h\left[ N \right]+\ldots+x\left[ 10 \right]\cdot h\left[ 2 \right]$$

$$= \sum_{i=2}^{N} p_{i}=1-Y\left[ i \right]$$

$$\ldots$$

$$Y\left[ 2N-1 \right]=1-Y\left[ 1 \right] i=2N+1$$

*Q.E.D*

**Theorem A3.1.2: Area invariance property**

Let $F\left( Y \right)=p[X]*h[X]$ be the BCDF of the discrete random variable $X=\{X_{1}, \ldots, X_{N}\}$, and $h\left( X \right)=\{1:X= X_{i}\}$ a N-dimensional unitary vector. Then $\int_{-\infty}^{\infty} F\left( Y \right)dy=N.$

*Proof*:

Let A$[F\left( Y \right)]$ denote the area of BCDF:

$$A\left[ F\left( Y \right) \right]=\sum_{i=-\infty}^{\infty} F\left( Y_{i} \right)= \sum_{i=1}^{2N-1} F{(Y}_{i})$$

Then

$$A\left[ F\left( Y \right) \right]=A\left[ p\left[ X \right]*h\left[ X \right] \right]=A\left[ p\left[ X \right] \right]\cdot A\left[ h\left[ X \right] \right]= \sum_{k=1}^{N} p\left[ X \right]\cdot\sum_{k=1}^{N} h\left[ X \right]=N$$

This can be proved by:

$$A\left[ F\left( Y \right) \right] = \sum_{i=1}^{2N-1} F\left( Y_{i} \right)= \sum_{i=1}^{2N-1} p\left[ X_{i} \right]*h[X_{i}]$$

$$=\sum_{i=1}^{2N-1} \sum_{k=1}^{N} p\left[ X_{k} \right]\cdot h\left[ i+1-k \right]$$

$$=\sum_{k=1}^{N} p\left[ X_{k} \right]\sum_{i=1}^{2N-1} h\left[ i+1-k \right]$$

$$=\sum_{k=1}^{N} p\left[ X_{k} \right]\sum_{i=1}^{2N-1} h\left[ i+\tau\right] \tau=1-k$$

Since the area $A\left[ h[X] \right]=A[h\left[ i+\tau\right]]$ (do not change with a shift in τ). It follows that:

$$A\left[ F\left( Y \right) \right]= \sum_{k=1}^{N} p[X_{k}]\sum_{k=1}^{N} h\left[ X_{k} \right]=N$$

*Q.E.D*

# **A3.2: Bilateral Cumulative Distribution Function Autocorrelation**

Let denote $\hat{F}\left( Y \right)$ the folding sequence of $F(Y)$:

$$\hat{F}\left( Y_{i} \right)=\left\{ \begin{aligned} F\left( Y_{N} \right) i=1 \\ \\ F\left( Y_{N-i+1} \right) i=\{2,.. , N\} \end{aligned} \right.$$

Then the autocorrelation function of $F(Y)$ is given by the following formula:

$$\boldsymbol{R}_{\boldsymbol{f}}\left( \boldsymbol{W} \right)=r_{f}\left( w_{i} \right)=F\left( Y \right)*\hat{F}\left( Y \right)= \sum_{k=1}^{N} F\left[ Y_{k} \right]\cdot\hat{F}\left[ i+1-k \right] i \in\{1, \ldots, 4N-3\}$$

Since the maximum value of the autocorrelation function occurs when the sequence correlates perfectly with itself with zero delay and the symmetry property, we can use the following relations:

$$r_{f}\left( w_{i} \right)=\left\{ \begin{aligned} \sum_{k=1}^{N} F\left[ Y_{k} \right]\cdot\hat{F}\left[ i+1-k \right] i \in\{1, \ldots, 2N-1\} \\ \\ r_{f}\left( w_{4N-2-i} \right) i \in\{2N, \ldots, 4N-3\} \end{aligned} \right.$$

It follows that the maximum occurs for $i=2N-1.$

Let denote $\delta(X)$ the singleton distribution of the random variable $X=\{X_{1}, \ldots, X_{N}\}$ :

$$\delta\left( X \right)=\left\{ \begin{aligned} 1 X_{i} i \in\left\{ 1, \ldots, N \right\} \\ \\ 0 X_{j} j \neq i \end{aligned} \right.$$

Then the autocorrelation function of $\delta(X)$, is given by the following formula:

$$\boldsymbol{R}_{\boldsymbol{\delta}}\left( \boldsymbol{W} \right)≝r_{\delta}\left( w_{i} \right)=\left\{ \begin{aligned} 0 i \in\{1, \ldots, N-1\} \\ \\ i-N+1 i \in\{N, \ldots, 2N-1\} \\ \\ r_{f}\left( w_{4N-2-i} \right) i \in\{2N, \ldots, 4N-3\} \end{aligned} \right.$$

The BCDFA is a symmetric function that preserves the variance of the pdf. See **theorem A3.2.1**

For the parametric formula of the minimum and maximum value of the BCDFA see **theorem A3.2.2**

**Theorem A3.2.1: variance BCDFA**

Let $R_{f}(W)$ be the BCDFA of the random variable $X=\left\{ X_{1},\ldots, X_{N} \right\}=\{1,2,\ldots,N\}$ and $h\left( X \right)=\{1:X= X_{i}\}$ a sequence of unit impulses spaced at unit interval. Let’s denote $\sigma_{Rf}^{2}, \sigma_{h}^{2} and \sigma_{x}^{2}$ the variance of the BCDFA, the impulses sequence and the probability distribution respectively. Then:

$$\sigma_{Rf}^{2}=k+2\sigma_{x}^{2} : k= \frac{N^{2}-1}{6}$$

*Proof*:

$$h\left( X_{i} \right)=1 i=1, \ldots, N$$

$$\sigma_{h}^{2}=E\left[ X^{2} \right]-\left( E\left[ X \right] \right)^{2} E\left[ X \right]=\frac{1}{N}\sum_{i=1}^{N} X_{i}=\frac{1}{N}\sum_{i=1}^{N} i=\frac{N+1}{2}$$

$$\sigma_{h}^{2}=\frac{(N+1)(2N+1)}{6}-\left( \frac{N+1}{2} \right)^{2} E\left[ X^{2} \right]=\frac{1}{N}\sum_{i=1}^{N} X_{i}=\frac{1}{N}\sum_{i=1}^{N} i^{2}=\frac{(N+1)(2N+1)}{6}$$

$$\sigma_{h}^{2}=\frac{N^{2}-1}{12}$$

It is known that the variance $\sigma_{X}^{2}$, which is a measure of spread of the probability distribution $p(x)$, is increased under convolution $h(x)$ according to the result: $\sigma_{p[X]*h[X]}^{2}=\sigma_{h}^{2}+\sigma_{X}^{2}$.

It follows that the BCDF of x (i.e. F(Y)) has variance: $\sigma_{F(Y)}^{2}=\sigma_{h}^{2}+\sigma_{X}^{2}$.

Similarly, the variance of the BCDFA is given by the formula:

$$\sigma_{Rf}^{2}= \sigma_{F(Y)}^{2}+\sigma_{F(Y)}^{2}=2\sigma_{F(Y)}^{2}\to\sigma_{F(Y)}^{2}=\frac{\sigma_{Rf}^{2}}{2}$$

It follows that:

$$\frac{\sigma_{Rf}^{2}}{2}=\sigma_{h}^{2}+\sigma_{X}^{2} \to\sigma_{Rf}^{2}=2\left[ \left( \frac{N^{2}-1}{12} \right)+\sigma_{X}^{2} \right]\to\sigma_{Rf}^{2}=k+2\sigma_{x}^{2} k= \frac{N^{2}-1}{6}$$

*Q.E.D*

**Theorem A3.2.2:**

Let $R_{f}(W)$ be the autocorrelation function of the random variable $X=\{X_{1},\ldots, X_{N}\}$. Then:

$$\max\left\{ R_{f}\left( 2N-1 \right) \right\}=N :\delta\left( X \right)=\left\{ p_{i}=1 \wedge p_{j}=0 \forall j\neq i \right\} i.e. singleton$$

$$\min\left\{ R_{f}\left( 2N-1 \right) \right\}=\frac{N-1}{2}+1 : \rho\left( X \right)=\left\{ p_{1}=p_{N}=\frac{1}{2} \wedge p_{j}=0 \forall j\neq1,N \right\}$$

*Proof*:

Based on theorem A2.2 and the definition of the BCDF (A2):

$$\int_{-\infty}^{\infty} R_{f}\left( w \right)dw= {\left[ F(Y) \right]^{2}=N}^{2}$$

For sake of simplicity, let’s denote $F\left( Y_{i} \right)=x_{i}$. It follows that:

$$N^{2}=\sum_{i=1}^{N} x_{i}^{2}+2x_{1}\left( \sum_{i=2}^{2N-1} x_{i} \right)+\ldots+2x_{j}\left( \sum_{i=j+1}^{2N-1} x_{i} \right)+\ldots+2x_{N}\left( \sum_{i=N+1}^{2N-1} x_{i} \right)+\ldots+2x_{N-1}x_{N}$$

Substituting the following relations:

$$\sum_{i=1}^{2N-1} x_{i}=N \to\sum_{i=j+1}^{2N-1} x_{i}=N-\sum_{i=1}^{j} \left( 1-x_{j} \right) \wedge x_{N}=\sum_{i=1}^{N} p_{i}=1 \wedge x_{i}=1-x_{k}\left\{ \begin{aligned} i\in\{1,..,2N-1\} \\ k \in\{1,\ldots,N-1\} \end{aligned} \right.$$

$$R_{f}\left( 2N-1 \right)=\sum_{i=1}^{N} x_{i}^{2}$$

It follows that:

$$R_{f}\left( 2N-1 \right)=2\sum_{i=1}^{N-1} x_{i}^{2}-2\sum_{i=1}^{N-1} x_{i}+N$$

The autocorrelation function of the BCDF is therefore a conic. In order to find the maximum and minimum value of the autocorrelation function, it is convenient to reduce the conic in a canonical form:

$$\left\{ \begin{aligned} x_{i}=\gamma_{i}+c_{i} \\ \\ x_{i}\leq x_{j} j>i \end{aligned} \right.$$

$$R_{f}\left( 2N-1 \right)=2\sum_{i=1}^{N-1} \gamma_{i}^{2}+\sum_{i=1}^{N-1} \gamma_{i}\left( -2+4c_{i} \right)+\lambda: \lambda=2\sum_{i=1}^{N-1} c_{i}^{2}-2\sum_{i=1}^{N-1} c_{i}+N$$

In order to eliminate the linear terms and operate the translation to the origin, we consider the following conditions:

$$-2+4c_{i}=0\to c_{i}=\frac{1}{2} Therefore \lambda=\frac{N-1}{2}+1$$

$$\left\{ \begin{aligned} max/\min f\left( \gamma\right)=2\sum_{i=1}^{N-1} \gamma_{i}^{2}+\frac{N-1}{2}+1 \\ \\ \gamma_{i}\leq\gamma_{j} j>i \\ \\ \gamma_{i}\in[0 \frac{1}{2}] \end{aligned}\underset{\Rightarrow}{} \left\{ \begin{aligned} \max f\left( \gamma^{*} \right)=N \wedge\gamma_{i}^{*}=\frac{1}{2} \forall i \\ \\ \min f\left( \gamma^{*} \right)=\frac{N-1}{2}+1 \wedge\gamma_{i}^{*}=0 \forall i \end{aligned} \right. \right.$$

Since $Y_{i}=\gamma_{i}+c_{i}$:

$$\max\left\{ R_{f}\left( w \right) \right\}=N Y_{i}^{*}=\left\{ \begin{aligned} 1 i=1,\ldots, N \\ \\ 0 otherwise \end{aligned} \to\delta\left( X \right)=\left\{ \begin{aligned} 1 i=1 \\ \\ 0 otherwise \end{aligned} \right. \right.$$

$$\min\left\{ R_{f}\left( w \right) \right\}=\frac{N-1}{2}+1 Y_{i}^{*}=\left\{ \begin{aligned} \frac{1}{2} i\neq N \\ \\ 1 i=N \end{aligned} \to\rho\left( X \right)=\left\{ \begin{aligned} \frac{1}{2} i=1,N \\ \\ 0 otherwise \end{aligned} \right. \right.$$

These two distributions correspond to the minimum and maximum variance of a bounded probability distribution:

$$\sigma^{2}\left( \delta\left( X \right) \right)= 0 < \sigma^{2}\left( P\left( X \right) \right)< \left( N-\frac{N+1}{2} \right)\left( \frac{N+1}{2}-1 \right) = \sigma^{2}(\rho\left( X \right))$$

It’s worth noting that in the case N=2 $\rho\left( X \right)=U(X)$.

***Q.E.D***

# **A3.3: DI-JSD**

Let $R_{f}(W)$ and $R_{\delta}(W)$ be the autocorrelation functions of the random variable $X=\{X_{1}, \ldots, X_{N}\}$ and the impulse distribution $\delta(X)$ [see A3].

Based on the area invariance property theorem A3.1.2

$$\int_{-\infty}^{\infty} R_{f}\left( w \right) dw= \int_{-\infty}^{\infty} R_{\delta}\left( W \right)dw=A\left[ F\left( Y \right) \right]\cdot A\left[ F\left( Y \right) \right]= N^{2}$$

It follows that the normalized sequences are:

$\hat{R_{f}}\left( W \right)=\frac{1}{N^{2}} \cdot R_{f}\left( W \right)$ and $\hat{R_{\delta}}\left( W \right)=\frac{1}{N^{2}} \cdot R_{\delta}\left( W \right)$

For sake of simplicity let denote $P\left( W \right)=\hat{R_{f}}\left( W \right)$ and $Q\left( W \right)=\hat{R_{\delta}}\left( W \right)$. Then, based on the Jensen-Shannon Divergence (JSD), we propose the following divergence measure:

$$\boldsymbol{DI}\left( \boldsymbol{X} \right)≝2\cdot JSD(P |\left| Q \right)=D(P|\left| M \right)+D(Q||M)$$

Where:

$M=\frac{1}{2}\left( P+Q \right)\to M\left( w_{i} \right)= \frac{1}{2}(P\left( w_{i} \right)+Q(w_{i})$) $0\leq DI\left( X \right)<1$

$$D(P|\left| M \right)= \sum_{w=1}^{4N-3} P\left( w \right)\cdot\log_{2} \frac{P\left( w \right)}{M\left( w \right)}= \sum_{w=1}^{4N-3} P(w)\cdot\left[ \log_{2} P\left( w \right)- \log_{2} M(w) \right]$$

$$D(Q|\left| M \right)= \sum_{w=1}^{4N-3} Q\left( w \right)\cdot\log_{2} \frac{Q\left( w \right)}{M\left( w \right)}= \sum_{w=1}^{4N-3} Q(w)\cdot\left[ \log_{2} Q\left( w \right)- \log_{2} M(w) \right]$$

Since $\lim_{w\to0^{+}} w\cdot\log w=0$ , we assume $\log0$ = 0.

**Theorem A3.3.1: Divergence index bound property**

Let DI(X) be the divergence index of the random variable $X=\{X_{1},\ldots,X_{N}\}$. Then the DI(X) is a bounded metric such that:

${\arg Max \atop P(x)}${DI(X)} =$\rho(x)$ $\rho\left( x \right)=\{p_{1}=p_{N}=\frac{1}{2} \wedge p_{j}=0 : j\neq1,N\}$

*Proof:*

Let $R_{fp}$ and $\tau_{0}$ be the autocorrelation function of the BCDF and its central coordinate (i.e. $\tau_{0}=2N-1$). Then we introduce the autocorrelation width as:

$$W_{Rfp}=\frac{\int_{-\infty}^{\infty} R_{fp}(w)}{R_{f}(\tau_{0})}=\frac{N^{2}}{R_{f}(\tau_{0})}$$

That is the area of the autocorrelation function divided by its central ordinate. Based on theorem A3.2.2, it follows that:

${\arg Max \atop P(x)}${$W_{Rfp}\}=\delta(x)$ ${\arg min \atop P(x)}${$W_{Rfp}\}=\rho(x)$

Then:

$$W_{Rf}\left( \delta\left( x \right) \right)=N$$

Since $R_{\delta}\left( w_{i} \right)=0 i \in\{1,\ldots,N-1\}$ then:

$$\int_{-\infty}^{\infty} R_{\delta}\left( w \right)dw= \int_{\tau_{0}-W_{Rf}+1}^{\tau_{0}+W_{Rf}-1} R_{\delta}\left( w \right)=\sum_{i=N}^{3N-2} r_{\delta}\left( w_{i} \right)=N^{2}$$

We therefore define as a measure of compactness for certain shapes of distribution:

$$S(N)=\frac{\sum_{i=N}^{3N-2} r_{fp}(w)}{N^{2}}=\left\{ \begin{aligned} \max\left\{ S \right\}=1 \leftrightarrow P\left( X \right)=\delta\left( X \right) : N>1 \\ \\ \min\left\{ S \right\}=\frac{3N+1}{4N} \leftrightarrow P\left( X \right)=\rho\left( X \right): \end{aligned} \right.$$

The minimum value can be easily computed, considering the autocorrelation of the BCDF $\rho\left( x \right).$

$$R_{\boldsymbol{f}}\left( \rho\left( X \right) \right)=\left\{ \begin{aligned} \frac{i}{4} i\in\{1,\ldots, N-1\} \\ \\ \frac{1}{2}+\frac{i}{4} i\in\left\{ N,\ldots, 2N-2 \right\} \\ \\ \frac{N+1}{2} i=2N-1 \end{aligned} \right.$$

$$\sum_{i=N}^{3N-2} R_{f}\left( w_{i} \right)=2\sum_{i=N}^{2N-2} r_{f}\left( w_{i} \right)+\frac{N+1}{2}$$

$$2\sum_{i=N}^{2N-2} r_{f}\left( w_{i} \right)=2\sum_{i=N}^{2N-2} \left( \frac{1}{2}+\frac{i}{4} \right)=\sum_{i=N}^{2N-2} 1+\sum_{i=N}^{2N-2} \frac{i}{2}=\left( N-1 \right)+\frac{(2N-2)(2N-1)}{4}-\frac{\left( N-1 \right)N}{4}$$

$$\sum_{i=N}^{3N-2} R_{f}\left( w_{i} \right)=\frac{N(3N+1)}{4} \underset{\Rightarrow}{} S_{\rho}=\frac{3N+1}{4N}$$

It follows that:

$$\lim_{N\to\infty} S_{\rho}(N)=\lim_{N\to\infty}\frac{3N+1}{4N}\cong\frac{3}{4}=0.75 \wedge\lim_{N\to1} S_{\rho}(N)=1 \to DI\left( \rho\left( x \right) \right)=0$$

The Divergence index is therefore a bounded metric. ***Q.E.D***

# **A3.4: Value validity HI**

To determine the condition for an HI to have Value validity, we shall use the two parameters $(\lambda,\mu)$ distribution, where the parameter $\mu$ reflects the polarization or dispersion of the distribution for a given value of $\lambda$. Thus, instead of $P_{n}^{\lambda}$ we shall use:

$$P_{n}^{\lambda,\mu}=\left[ \left( 1-\frac{\mu}{2} \right)\cdot\left( 1-\lambda\right)+\frac{\lambda}{n},\frac{\lambda}{n},\cdots,\frac{\mu}{2}\left( 1-\lambda\right)+\frac{\lambda}{n} \right] : \lambda,\mu\in\left[ \begin{matrix} 0 & 1 \end{matrix} \right] n>3 (3)$$

which reduces to equation $(2)$ for $\mu=0$ and to:

$$P_{n}^{0,\mu}=\left( 1-\frac{\mu}{2},0,\cdots,0,\frac{\mu}{2} \right)= \mu\cdot P_{n}^{0,1}+(1-\mu)\cdot P_{n}^{0} : \mu\in\left[ \begin{matrix} 0 & 1 \end{matrix} \right] (4)$$

for $\lambda=0.$ For example, the two-point extreme distribution $P_{n}^{0,1}$ is the most dispersed ($\mu=1)$ and the least heterogeneous $(\lambda=0)$ configuration. On the other hand, the $P_{n}^{0}$ is the least dispersed and heterogeneous distribution $(i.e. HI(P_{n}^{0})=1)$. Lastly, the uniform distribution $P_{n}^{1}$ is the only distribution where $\lambda=1$ and therefore the most heterogeneous $(i.e. HI(P_{n}^{1})=0 \forall\mu)$. It is assumed of course, that $HI(P_{n}^{\lambda,\mu})$ is strictly decreasing in μ for any given λ. This result is a natural consequence of the simple aversion to median-preserving spreads property.

In this framework, we should expect that a valid HI will be closer to the CI curve for smaller value of the parameter μ and gives reasonably high values for the two-point extreme distribution. This insight leads to the problem of choosing a HI which should vary as little as possible from a selected target function and be not too far away from the CI curve. The task is therefore to find a Value validity condition which incorporates the idea that a well behaved or “smoother” HI is more appropriate than a “rougher” HI. This requirement implies that the HI is bounded above by the values assigned to a target function and below by a minimum curve. These functions set the boundaries of the feasible HI measures and identify a class of slowing decreasing functions or dual Schwartz functions [8] in the $(\lambda,\mu)$ space. This means that small changes in the distribution vector $P^{\lambda,\mu}$ should not lead to abrupt changes in the value of the index. This behavior does not overstate evenness and give acceptable values when the distributions have large variance. In this way, the upper and lower bound functions identify a feasible region for the HI. Then, the HI has Value validity if it satisfies the following two conditions:

$$\left\{ \begin{aligned} \left( \boldsymbol{C}\boldsymbol{1} \right): L\left( D,HI \right)=\iint\left( 1-\mu\right)\cdot HI\left( P_{n}^{\lambda,\mu} \right) d\lambda d\mu-0.5 \geq0 \\ \\ \left( \boldsymbol{C}\boldsymbol{2} \right): L\left( D,HI \right)\leq L\left( D,S \right)= \iint\left( 1-\mu\right)\left( 1-\lambda\right)\left[ 1-\frac{\left( n+1 \right)\mu}{4\left( n-1 \right)} \right]d\lambda d\mu-0.5 \\ \end{aligned} \right.$$

(**C1**): this condition is based on a loss function $L(D,HI)$ which indicates how far away is the HI from the lower function. More precisely, the value of this function must be positive.

(**C2**): the value of the loss function $L(D,HI)$ must be less than or equal to the loss function for the target function $L(D,S)$.

# **A3.5: CI and True Diversity**

The true diversity $s$ gives the number of equally abundant categories needed to give a particular value of a concentration index.

The conversion of $s$ to any concentration measure, such as the CI, can be easily obtained for a countable number of categories: calculate the CI for s equally abundant categories (s bins with a frequency $1/s$) and assign that value to $s$. But how is possible to link the CI to any value of $s$?

As discussed in section A3 the CI satisfies the Value validity property and therefore it follows a straight line that basically is an arithmetic progression. In a similar way, the CI value can be mapped to the true diversity number in a linear manner. Consequently, the direct and inverse formula are:

$$s=D\left( n,CI \right)=n-n\left( n-1 \right)\cdot CI CI\left( n,s \right)=\frac{n-s}{n-1} CI\in\left[ 0 1 \right] \wedge s\in[1 n]$$

Where:

- $CI : the concentration Index value$
- $n : the total number of categories in the histogram$

# **Appendix B: Location Index**

An important task of spatial data analysis consists in identifying a part of data which represents the typical features of the population living in a geographic area. Often the data points are assumed to vary around a centre that identifies a single value as representative of an entire distribution. As a result, we propose a new measure of central tendency, the “**Location Index**” (**LI**), that identifies the position of the bin in the distribution where the values are mostly concentrated (i.e. the values in the surrounding areas of the bin are noticeably higher than the others).

The basic idea is to map a nested family of sets around the location of the bin into a single number. If the distribution is univariate the set is a symmetric interval formed by a finite number of categories and the concentration value is given by summing up the likelihood of these regions. In this way, each location has a concentration value and the one with the maximum value corresponds to the point of the LI. For the definition of the LI see (**B1: Location Index**).

Moreover, the definition of the function used in the computation of the bin concentration value, the Bin Concentration Function (BCF), provides simple rules to determine the location of a distribution. For example, considering a decile distribution, the set of necessary and sufficient conditions to determine the LI value is given in Table 1.

| Location  Index | $\boldsymbol{p}\left[ \boldsymbol{i,j} \right]\boldsymbol{=}\sum_{\boldsymbol{k=i}}^{\boldsymbol{j}} \boldsymbol{p}_{\boldsymbol{k}}\boldsymbol{\cdot100}$ | |
| --- | --- | --- |
| Decile | $\geq50\%$ | $\leq50\%$ |
| 1 | $[1,1]$ | $[2,10]$ |
| 2 | $\left[ 1,2 \right]$ | $[3,10]$ |
| 3 | $\left[ 1,3 \right]$ | $[4,10]$ |
| 4 | $\left[ 1,4 \right]$ | $[5,10]$ |
| 5 | $\left[ 1,5 \right]$ | $[6,10]$ |
| 6 | $\left[ 1,6 \right]$ | $[7,10]$ |
| 7 | $\left[ 1,7 \right]$ | $[8,10]$ |
| 8 | $\left[ 1,8 \right]$ | $[9,10]$ |
| 9 | $\left[ 1,9 \right]$ | $[10,10]$ |
| 10 | $[10,10]$ | $[1,9]$ |

**Table 1 Location Index conditions**

In this table, the first and remaining columns indicate the LI and the deciles intervals that contain at least $50\%$ of the data and those with less or equal to $50\%$ of the samples. For example, the first and last row show that a distribution with a LI equal to one or ten have at least $50\%$ of the data in the first or last decile. Similarly, the value of this index for the other bins indicates the decile position separating the greater and lesser halves of a distribution.

Therefore, the LI turns out to be an equivalent form of the median, having a breakdown point of $50\%$: so long as no more than half the data are too much dispersed, the LI will not give an arbitrary large or small result. The basic difference of the LI in describing data compared to the median is that the outcome is always a member of the distribution and it is easier to calculate. In addition, since more than one value can be at the median level, this location parameter identifies a unique interval of values for which the conditions hold. Lastly, the LI definition can be generalized to a multivariate distribution.

Finally, Table 2 summarizes several of the properties of the central tendencies measures that have been discussed in the paper.

For the properties see Theorem B1.1 and lemma B1.1-5.

|  | Properties of Measures of Center | | | |
| --- | --- | --- | --- | --- |
|  | Mode | Median | Mean | LI |
| Level of measurement | Nominal or higher | Ordinal or higher | Metric usually | Ordinal or higher |
| Simple to understand | best | yes | yes | Yes |
| Easy to calculate | best | yes | yes | Yes |
| Algebraic | No | No | yes | Yes |
| Single valued | Not Always | yes | best | Not Always |
| Resistant to outliers | yes | yes | no | Yes |
| Generalize to  n-variables | no | no | yes | Yes |
| Easy interpretation for n variables | no | no | no | Yes |
| Equal to actual data values | yes | Not always | Not Always | Yes |
| Interpretation | Most typical value | Middle value | Average value  Center of mass | Set of middle values |
| Bad guess interpretation | Highest % accuracy | Closest to all scores | Minimized sum of squared deviations or signed deviation | Closest to all scores. Highest concentration |

Table 2 Central Tendency Measure Properties - Adaption of Central Tendency and variability [9]

# **B1: Location Index**

Let $I_{x_{0}}=[x_{m} x_{M}]$ be a closed bounded interval of the random variable $X=\left\{ x_{1}, \ldots, x_{N} \right\}\to\left| X \right|=N.$ Then $\forall x_{0}\in X \exists I_{x_{0}}: x_{m}\leq x_{0}\leq x_{M} \wedge x_{m},x_{0},x_{M} \in\{1, \ldots, N\}$. It follows that $\left| I_{x_{0}} \right|=M-m+1=b$. In the case of a symmetric closed bounded interval $b=2k+1.$

$$I_{x_{0},k}=\left[ x_{0}-k x_{0}+k \right] \to\left| I_{x_{0}} \right|=2k+1$$

Let’s denote the set of nested compact subset of $x_{0}$ with the following notation:

$$I_{x_{0}}=\bigcup_{k=0}^{\infty} I_{x_{0},k}\to\bigcap_{k=0}^{\infty} I_{x_{0},k}\neq\left\{ \emptyset\right\}\to X\subset I_{x_{0}}$$

Then, let’s define the functions $\varphi$(**Likelihood Central Region function)** and $\gamma$ (**Level Set Density function**).

$$\varphi\left( x_{0},k \right)=\sum_{i=x_{0}-k}^{x_{0}+k} P\left( X=x_{i} \right) : k \in\{0, \ldots, N-1\}$$

$$\gamma\left( x_{0},s \right)= \sum_{k=0}^{s} \sum_{i=x_{0}-k}^{x_{0+k}} P(X=x_{i})$$

Then the **Bin Concentration function** (BCF), is given by the following formula:

$$BCF=\gamma\left( x_{0} \right)=\gamma\left( x_{0},N-1 \right)=\sum_{k=0}^{N-1} \sum_{i=x_{0}-k}^{x_{0}+k} P(X=x_{i})$$

Let $\Gamma\left( X \right)=\{\gamma_{1},\ldots, \gamma_{i}, \ldots, \gamma_{N}\}$be the BCF vector of the random varaible X.

$$\gamma_{i}=\gamma\left( x_{i} \right)=\gamma\left( x_{i},N-1 \right)=\sum_{k=0}^{N-1} \sum_{i=x_{0}-k}^{x_{0}+k} P(X=x_{i})$$

Then, the **Location Index** of this random variable is given by the formula:

$\lambda: \mathbb{R}^{N}\to N x N$ $\lambda=\left( \lambda_{1},\lambda_{2} \right). N=\{1, \ldots, N\}$

$$\lambda_{1}=arg \min_{i\in N} \{ Max \gamma_{i} \in\Gamma\left( X \right)\}$$

$$\lambda_{2}=arg \max_{i\in N} \{ Max \gamma_{i} \in\Gamma\left( X \right)\}$$

**Theorem B1.1- Location index convergence property:** Let $\Phi_{x_{0}}=\{\varphi_{0}, \ldots, \varphi_{k}, \ldots, \varphi_{N-1}\}$ be a sequence in $X=\{x_{1}, \ldots, x_{N}\}$, such that:

$$\varphi_{k}=\varphi\left( x_{0},k \right)=\sum_{i=x_{0}-k}^{x_{0}+k} P(X=x_{i})$$

Then $\forall x_{0} \in X \exists k_{m}: \varphi\left( x,k \right)=1 \forall k\geq k_{m}$.

Proof:

Let $I_{x}$ and $I_{x0,X}$ be two collections of non empty closed bounded intervals in ℤ such that

$$I_{x}=\left[ x_{1} x_{N} \right] and I_{x0,X}=\bigcup_{k=0}^{N-1} I_{x_{0},k}$$

Since $\left| I_{x} \right|=N$ and $\left| I_{x_{0},X} \right|=2N-1$, it follows that:

$$\forall x_{0}\in X \exists k_{m}: I_{x}\subseteq\bigcup_{j=0}^{k} I_{x_{0},j} \forall k\geq k_{m} \to E=I_{x}\bigcap\bigcup_{j=0}^{k_{m}} I_{x_{0},j} \neq\{\emptyset\}$$

Then

$$\bigcup_{j=0}^{k} I_{x_{0},j}=E \bigcup\bar{E}$$

It follows that

$$E=X=\{x_{1}, \ldots, x_{N}\}\to\sum_{i=1}^{N} P\left( X=x_{i} \right)=1 \wedge\bar{E}=\bar{X}\to\sum_{i\in\bar{X}} P\left( X=x_{i} \right)=0$$

Therefore,

$$\varphi_{k_{m}}=\varphi\left( x_{0},k_{m} \right)=\varphi\left( x_{0},k \right)=\sum_{i=1}^{N} P\left( X=x_{i} \right)+\sum_{i\in\bar{X}} P\left( X=x_{i} \right)=1$$

$$\varphi\left( x_{0},k_{m} \right)=\sum_{i=x_{0}-k_{m}}^{x_{0}+k_{m}} P\left( X=x_{i} \right)=\sum_{i=x_{0}-k}^{x_{0}+k} P\left( X=x_{i} \right) \forall k \geq k_{m}$$

**Q.E.D**

**Lemma B1.1** Let $\varphi(j,k)$ be the *Likelihood Central Region Function* and $\gamma(x_{j})$ be the BCF of the random variable $X=\left\{ x_{1},\ldots, x_{N} \right\}$. Then the BCF is a linear convex combination such that:

$$\gamma\left( x_{j} \right)=\sum_{i=1}^{N} w_{i}\cdot p_{i} : w_{i}\in\{1,2,\ldots,N\}$$

$$\gamma\left( x_{j} \right)=\left\{ \begin{aligned} \sum_{i=0}^{N-1} \left( N-i \right)\cdot p_{i+1} j=1 \\ \\ \sum_{i=0}^{j-1} (i+N-j+1)\cdot p_{i+1}+\sum_{i=j}^{N-1} (N-i+j-1)\cdot p_{i+1} 1<j<N \\ \\ \sum_{i=0}^{N-1} (i+1)\cdot p_{i+1} j=N \end{aligned} \right.$$

*Proof:*

Let $j=1$, then the *Likelihood Central Region Function* is given by the following formula:

$$\varphi\left( 1,k \right)=\sum_{i=1-k}^{1+k} P\left( X=x_{i} \right) : k\in\{0,\ldots, N-1\}$$

Since $P\left( X=x_{i} \right)=0 \forall i<1,$ it follows that:

$$\varphi\left( 1,k \right)=\sum_{i=1}^{k} P\left( X=x_{i} \right)=\sum_{i=0}^{k-1} P(X=x_{i+1})=\sum_{i=0}^{k-1} p_{i+1} : k\in\{1, \ldots, N-1\}$$

Then the *Level Set Density Function* is given by the following equation:

$$\gamma\left( 1,s \right)=\sum_{k=1}^{s} \sum_{i=0}^{k-1} p_{i+1} : s\in\{1, \ldots, N\}$$

$$\gamma\left( 1,1 \right)=p_{1}$$

$$\gamma\left( 1,2 \right)={2\cdot p}_{1}+p_{2}$$

$$\vdots$$

$$\gamma\left( 1,s \right)={s\cdot p}_{1}+\left( s-1 \right)\cdot p_{2}+\ldots+\left( N-i \right)\cdot p_{i+1}+\ldots+p_{N}$$

Then for $s=N$

$$\gamma\left( 1,N \right)={N\cdot p}_{1}+\left( N-1 \right)\cdot p_{2}+\ldots+\left( N-i \right)\cdot p_{i+1}+\ldots+p_{N}$$

It follows that:

$$\gamma\left( 1,N \right)=\sum_{i=0}^{N-1} (N-i)\cdot p_{i+1}$$

*Q.E.D*

Let’s prove for $j=N$:

$$\varphi\left( N,k \right)=\sum_{i=N-k}^{N+k} P\left( X=x_{i} \right) : k\in\{0,\ldots, N-1\}$$

Since $P\left( X=x_{i} \right)=0 \forall i>N$, it follows that:

$$\varphi\left( N,k \right)=\sum_{i=N-k}^{N} P\left( X=x_{i} \right)=\sum_{i=N-k}^{N-1} P(X=x_{i+1})=\sum_{i=N-k}^{N-1} p_{i+1} : k\in\{1, \ldots, N\}$$

Then the *Level Density Function* is given by the following equation:

$$\gamma\left( N,s \right)=\sum_{k=1}^{s} \sum_{i=N-k}^{N-1} p_{i+1} : s\in\{1, \ldots, N\}$$

$$\gamma\left( N,1 \right)=p_{N}$$

$$\gamma\left( N,2 \right)={2\cdot p}_{N}+p_{N-1}$$

$$\vdots$$

$$\gamma\left( N,s \right)={s\cdot p}_{N}+\left( s-1 \right)\cdot p_{N-1}+\ldots+p_{N-s}$$

Then for $s=N$

$$\gamma\left( N,N \right)={N\cdot p}_{N}+\left( N-1 \right)\cdot p_{N-1}+\ldots+\left( N-i \right)\cdot p_{N-i}+\ldots+p_{1}$$

It follows that:

$$\gamma\left( N,N \right)=\sum_{i=0}^{N-1} (i+1)\cdot p_{i+1}$$

*Q.E.D*

Let $2\leq j\leq N-1$

The *Likelihood Central Region Function* is given by the following formula:

$$\varphi\left( j,k \right)=\sum_{i=k-1}^{k+1} P\left( X=x_{i} \right) : k\in\{1,\ldots, N\}$$

Since $P\left( X=x_{i} \right)=0 \forall i<1 \wedge i>N$. It follows from the previous results that:

$$\varphi\left( j,k \right)=\sum_{i=j-k}^{j-1} P\left( X=x_{i+1} \right)+\sum_{i=j}^{j+k-2} P\left( X=x_{i+1} \right) : k\in\left\{ 1,\ldots, N \right\}$$

$$=\sum_{i=j-k}^{j-1} p_{i+1}+\sum_{i=j}^{j+k-2} p_{i+1}$$

Then the *Level Set Density Function* is given by the following equation:

$$\gamma\left( j,s \right)=\sum_{k=1}^{s} \sum_{i=j-k}^{j-1} p_{i+1}+\sum_{k=2}^{s} \sum_{i=j}^{j+k-2} p_{i+1} : s\in\{1, \ldots, N\}$$

Let’s solve the first term:

$$\sum_{k=1}^{s} \sum_{i=j-k}^{j-1} p_{i+1}$$

$$\gamma\left( j,1 \right)=p_{j}$$

$$\gamma\left( j,2 \right)={2\cdot p}_{j}+p_{j-1}$$

$$\vdots$$

$$\gamma\left( j,s \right)={s\cdot p}_{j}+\left( s-1 \right)\cdot p_{j-1}+\ldots+p_{j-s}$$

Then for $s=N$

$$\gamma\left( j,N \right)={N\cdot p}_{j}+\left( N-1 \right)\cdot p_{j-1}+\ldots+p_{j-N}$$

Since $P\left( X=x_{i} \right)=0 \forall i<1$

$$\gamma\left( j,N \right)={N\cdot p}_{j}+\left( N-1 \right)\cdot p_{j-1}+\ldots+{(N-j+1)\cdot p}_{1}$$

It follows that:

$$\gamma\left( j,N \right)=\sum_{i=0}^{j-1} (i+N-j+1)\cdot p_{i+1}$$

The second term:

$$\sum_{k=2}^{s} \sum_{i=j}^{j+k-2} p_{i+1}$$

$$\gamma\left( j,2 \right)=p_{j+1}$$

$$\gamma\left( j,3 \right)={2\cdot p}_{j+1}+p_{j+2}$$

$$\vdots$$

$$\gamma\left( j,s \right)={(s-1)\cdot p}_{j}+\left( s-2 \right)\cdot p_{j-1}+\ldots+p_{j+s-1}$$

Then for $s=N$

$$\gamma\left( j,N \right)={(N-1)\cdot p}_{j}+\left( N-2 \right)\cdot p_{j-1}+\ldots+p_{j+s-1}$$

Since $P\left( X=x_{i} \right)=0 \forall i>N$

$$\gamma\left( j,N \right)={(N-1)\cdot p}_{j+1}+\left( N-2 \right)\cdot p_{j+2}+\ldots+{j\cdot p}_{1}$$

It follows that:

$$\gamma\left( j,N \right)=\sum_{i=j}^{N-1} (N-i+j-1)\cdot p_{i+1}$$

***Q.E.D***

**Corollary B1.1**: Let $\mu_{U}$ be the expected value of the uniform distribution and $m=\left\lfloor\mu_{u} \right\rfloor$ be the rounded floor value. Then: $W=\{1, \ldots,N\}$

$$arg \min_{w_{i}} \left\{ x_{j} \right\}=\left\{ \begin{aligned} j j\in[1 m] \\ \\ N-j+1 j\in[m+1 N] \end{aligned} \right. \wedge arg\max_{w_{i}} \left\{ x_{j} \right\}=N$$

*Proof:*

$$arg\max_{w_{i}} \left\{ x_{j} \right\}=N$$

For $j=1$

$$\left\{ \begin{aligned} \max f(i) \\ \\ i\in\{0,...,N-1\} \end{aligned}\to\max\left( N-i \right)=N i^{*}=0 \right.$$

For $1<j<N$

$$max\left\{ \begin{aligned} \max\left( i+N-j+1 \right), max(N-i+j-1) \\ \\ i\in\left\{ 0,\ldots, j-1 \right\}, i \in\{j, \ldots,N-1\} \end{aligned} \right.\left. \begin{aligned} \\ \\ \\ \end{aligned} \right\}=N i^{*}=j-1$$

For $j=N$

$$\left\{ \begin{aligned} \max f(i) \\ \\ i\in\{0,...,N-1\} \end{aligned}\to\max\left( i+1 \right)=N i^{*}=N-1 \right.$$

$$arg \min_{w_{i}} \left\{ x_{j} \right\}$$

For $j=1$

$$\left\{ \begin{aligned} \min f(i) \\ \\ i\in\{0,...,N-1\} \end{aligned}\to\min\left( N-i \right)=1=j i^{*}=N-1 \right.$$

For $1<j<N$

$$min\left\{ \begin{aligned} \min\left( i+N-j+1 \right), min(N-i+j-1) \\ \\ i\in\left\{ 0,\ldots, j-1 \right\}, i \in\{j, \ldots,N-1\} \end{aligned} \right.\left. \begin{aligned} \\ \\ \\ \end{aligned} \right\}=\left\{ \begin{aligned} j j\in\left\{ 2,\ldots,m \right\} i^{*}=N-1 \\ \\ N-j+1 j\in\left\{ m+1 N \right\} i^{*}=0 \end{aligned} \right.$$

For $j=N$

$$\left\{ \begin{aligned} \min f(i) \\ \\ i\in\{0,...,N-1\} \end{aligned}\to\min\left( i+1 \right)=1 i^{*}=0 \right.$$

$$\min\left( i+1 \right)=min(i+N-j+1)\underset{\to}{j=N} 1$$

***Q.E.D***

**Lemma B1.2:** Let $\Gamma(X)=[\gamma_{1}, \ldots, \gamma_{i}, \ldots, \gamma_{N}]$ be the BCF vector of the random variable X

and $m=\left\lfloor\mu_{u} \right\rfloor$. Then

$$arg \max_{\gamma_{i}} \left\{ \Gamma(X) \right\}=\gamma_{j}\leftrightarrow\gamma_{j}:\left\{ \begin{aligned} \sum_{i=1}^{j-1} p_{i}\leq\frac{1}{2} \\ \\ \sum_{i=j+1}^{N} p_{i}\leq\frac{1}{2} \end{aligned} \wedge\left\{ \begin{aligned} \sum_{i=1}^{j} p_{i}\geq\frac{1}{2} j\in[1 m] \\ \\ \sum_{i=j}^{N} p_{i}\geq\frac{1}{2} j\in[m+1 N] \end{aligned} \right. \right.$$

*Proof:*

According to the *Corollary B1.1* :

$$\gamma\left( x_{j} \right)=\sum_{i=1}^{N} w_{i}\cdot p_{i} \left\{ \begin{aligned} w_{i} \in\left[ 1 N \right] : j\in[1 m] \\ \\ w_{i} \in\left[ N-j+1 N \right] : j\in[m+1 N] \end{aligned} \right.$$

Let’s prove

$$arg \max_{\gamma_{i}} \left\{ \Gamma\left( X \right) \right\}=\gamma_{1}=x_{1} \leftrightarrow p_{1}\geq\frac{1}{2}$$

It follows that:

$$\gamma\left( x_{1} \right)=N\cdot p_{1}+\left( N-1 \right)\cdot p_{2}+\ldots+\left( N-i \right)\cdot p_{i+1}+\ldots+p_{N}$$

$$\gamma\left( x_{1} \right)=(N-1)\cdot p_{1}+N\cdot p_{2}+\ldots+\left( N-i+1 \right)\cdot p_{i+1}+\ldots+2\cdot p_{N}$$

So $\gamma\left( x_{1} \right)\geq\gamma\left( x_{2} \right)\leftrightarrow\gamma\left( x_{1} \right)-\gamma(x_{2})\geq0$

For sake of simplicity let’s denote $P=[p_{1}, \ldots,p_{N}]$, then:

$$arg\max_{P} \left\{ \gamma\left( x_{1} \right) \right\}=\{p_{1}=1 \wedge p_{i}=0 \forall i \neq1\}$$

$$arg\min_{P} \left\{ \gamma\left( x_{1} \right) \right\} : \gamma(x_{1})\geq\gamma(x_{2})$$

$$Let p_{1}=\frac{1}{2}\to\gamma\left( x_{1} \right)=\left\{ \begin{aligned} \hat{\gamma_{1}}=\frac{N}{2}+\sum_{i=1}^{N-1} (N-i)\cdot p_{i+1} \\ \\ \sum_{i=2}^{N} p_{i}=\frac{1}{2} \end{aligned} \right.$$

It follows:

$$max \hat{\gamma_{1}}=\frac{N}{2}+\frac{N-1}{2} : p_{2}=\frac{1}{2}$$

$$\gamma\left( x_{2} \right)=\left\{ \begin{aligned} \hat{\gamma_{2}}=\frac{N-1}{2}+N\cdot p_{2}+\sum_{i=2}^{N-1} (N-i+1)\cdot p_{i+1} \\ \\ \sum_{i=2}^{N} p_{i}=\frac{1}{2} \end{aligned} \right.$$

$$max \hat{\gamma_{2}}=\frac{N-1}{2}+\frac{N}{2} : p_{2}=\frac{1}{2}$$

It follows that $\gamma\left( x_{1} \right)\geq\gamma\left( x_{2} \right)\leftrightarrow p_{1}\geq\frac{1}{2}\to\gamma\left( x_{1} \right)\geq\gamma\left( x_{j} \right)\leftrightarrow p_{1}\geq\frac{1}{2} \forall j\in\{2, ..,N\}$

Assume

$$arg \max_{\gamma_{i}} \left\{ \Gamma\left( X \right) \right\}=\gamma_{2}=x_{2}$$

Then

$$\gamma\left( x_{2} \right)\geq\gamma\left( x_{1} \right) \leftrightarrow p_{2}\geq p_{1}$$

$$\gamma\left( x_{2} \right)\geq\gamma\left( x_{3} \right) \leftrightarrow{p_{1}+p}_{2}\geq\frac{1}{2}$$

It follows that:

$$\gamma\left( x_{2} \right)\geq\gamma\left( x_{j} \right) \leftrightarrow{p_{1}+p}_{2}\geq\frac{1}{2} \forall j\in\{3,\ldots,N\}$$

Therefore, for $j \in[1, \ldots,m]$

$$\gamma\left( x_{j} \right)\geq\gamma\left( x_{i} \right) \leftrightarrow\sum_{i=1}^{j-1} p_{i}\leq\frac{1}{2} \wedge\sum_{i=1}^{j} p_{i}\geq\frac{1}{2} \wedge\sum_{i=j+1}^{N} p_{i}\leq\frac{1}{2}$$

Similarly, for $j \in[m+1, \ldots, N]$

$$\gamma\left( x_{j} \right)\geq\gamma\left( x_{i} \right) \leftrightarrow\sum_{i=1}^{j-1} p_{i}\leq\frac{1}{2} \wedge\sum_{i=j}^{N} p_{i}\geq\frac{1}{2} \wedge\sum_{i=j+1}^{N} p_{i}\leq\frac{1}{2}$$

***Q.E.D***

**Lemma B1.3** Let $\lambda=(\lambda_{1},\lambda_{2})$ be the LI of the random variable $X=\{x_{1}, \ldots, x_{N}\}$ and $P\left( X \right)=\left\{ x_{1},\ldots,x_{N} \right\}$ its probability mass function. Then:

$$arg\max_{P(X)} \{\lambda\left( P\left( X \right) \right\}=\delta\left( X \right) \to Max\left\{ \lambda\left( P\left( X \right) \right) \right\}=N$$

$$arg\min_{P(X)} \{\lambda\left( P\left( X \right) \right\}=\rho\left( X \right) \to min\left\{ \lambda\left( P\left( X \right) \right) \right\}=\frac{N+1}{2}$$

*Proof:*

Since the BCF is a linear convex combination:

$$\gamma\left( x_{j} \right)=\sum_{i=1}^{N} w_{i}\cdot p_{i} : arg\max_{w_{i}} \left\{ \gamma\left( x_{j} \right) \right\}=N=w_{j}$$

It follows that the maximum value is attained when the coefficients $p_{i}$ of the polynomial are all set to zero execpt for $p_{j}=1.$ In other words:

$$Max\left\{ \gamma\left( x_{j} \right) \right\}=N \leftrightarrow p_{j}=1 \wedge p_{i}=0 \forall i\neq j \to\lambda=(j,j)$$

On the other hand, the minimum value is attained when all the bins have exactly the same concentration value. This situation occurs when $p_{1}=p_{N}=0.5$.

$$\gamma\left( 1,N \right)= N\cdot p_{1}+\left( N-1 \right)\cdot p_{2}+\ldots+2\cdot p_{N-1}+p_{N}$$

$$\vdots$$

$$\gamma\left( j,N \right)=\left( N-j+1 \right)\cdot p_{1}+\ldots+\left( N-1 \right)\cdot p_{j-1}+N\cdot p_{j}+\ldots.+(j-1)\cdot p_{N-1}+j\cdot p_{N}$$

$$\vdots$$

$$\gamma\left( N,N \right)= p_{1}+2\cdot p_{2}+\ldots+(N-1)\cdot p_{N-1}+N\cdot p_{N}$$

Substituiting $p_{1}=p_{N}=0.5$.

$$\gamma\left( 1,N \right)= \frac{N}{2}+\frac{1}{2}=\frac{N+1}{2}$$

$$\gamma\left( 2,N \right)= \frac{(N-1)}{2}+1=\frac{N+1}{2}$$

$$\vdots$$

$$\gamma\left( j,N \right)=\frac{(N-j+1)}{2}+\frac{j}{2}=\frac{N+1}{2}$$

$$\vdots$$

$$\gamma\left( N,N \right)=\frac{(N-j)}{2}+\frac{(j+1)}{2}=\frac{(N+1)}{2} \to\lambda=(1,N)$$

***Q.E.D***

**Lemma B1.4:** Let $\lambda=(\lambda_{1},\lambda_{2})$ be the LI of the random variable $X=\left\{ x_{1},\ldots,x_{N} \right\}$ and $C=\gamma(\lambda)$ be the Bin concentration value. Then the normalized value is given by the following formula:

$$Bin Compactness: C_{\lambda}=\frac{2C-N-1}{N-1} Bin Compactness Deviation:\bar{C_{\lambda}}=\frac{2(N-C)}{N-1}$$

*Proof:*

According to Lemma B1.3:

$$Max\{\lambda\left( P\left( X \right) \right\}=N min\{\lambda\left( P\left( X \right) \right\}=\frac{(N+1)}{2}$$

It follows that:

$$C_{\lambda}=\frac{C-min\{\lambda(P\left( X \right)\}}{Max\{\lambda(P\left( X \right)\}-min\{\lambda(P\left( X \right)\}}=\frac{2C-N-1}{N-1}$$

It follows that

$$C_{\lambda}=1 \leftrightarrow P\left( X \right)=\delta\left( X \right) \to C=N$$

$$C_{\lambda}=0 \leftrightarrow P\left( X \right)=\rho\left( X \right) \to C=\frac{N+1}{2}$$

As for the Bin compactness deviation:

$$\bar{C_{\lambda}}=1-C_{\lambda}=\frac{2(N-C)}{N-1}$$

***Q.E.D***

**Lemma B1.5**: Let ${\gamma(x}_{j})$ be the BCF of the random variable $X=\{x_{1},\ldots, x_{j}, ..,x_{N}\}$ and ${\delta(x}_{j})$ be the average absolute deviation. Then:

$$arg \max_{j} \{{\gamma(x}_{j})\} = arg \min_{j} \{{\delta(x}_{j})\} j\in\{1,\ldots,N\}$$

*Proof:*

For sake of simplicity let’s dentoe:

$$\hat{\delta}\left( x_{j} \right)=\sum_{i=1}^{N} P(X=x_{i})\cdot|i-j|$$

It follows that:

$${\delta(x}_{j})=\frac{1}{N}\hat{\delta}\left( x_{j} \right)$$

$$\hat{\delta}\left( x_{j} \right)=\left\{ \begin{aligned} \sum_{i=0}^{N-1} i\cdot p_{i+1} j=1 \\ \\ \sum_{i=0}^{j-1} \left( j-1-i \right)\cdot p_{i+1}+\sum_{i=j}^{N-1} (i-j+1)\cdot p_{i+1} 1<j<N \\ \\ \sum_{i=0}^{N-1} \left( N-1-i \right)\cdot p_{i+1} j=N \end{aligned} \right.$$

Let $j=1\to\hat{\delta}\left( x_{1} \right)=\sum_{i=0}^{N-1} i\cdot p_{i+1}$

$$\gamma\left( x_{1} \right)=N\cdot p_{1}+\left( N-1 \right)\cdot p_{2}+\ldots+\left( N-1+1 \right)\cdot p_{i}+\ldots+p_{N}$$

$$=N\cdot\sum_{i=1}^{N} p_{i}-\sum_{i=0}^{N-1} i\cdot p_{i+1}=N-\hat{\delta}\left( x_{1} \right)$$

Let $p_{i}=P(X=x_{i})$ and $P=\{p_{1},\ldots,p_{i},\ldots,p_{N}\}$ be the mass probability of the random variable $X=\{x_{1},\ldots,x_{i},\ldots,x_{N}\}$. Then, since $\max f\left( x \right)=\min f(-x)$, it follows that:

$\arg\max_{P} \left\{ \gamma\left( x_{1} \right) \right\}=arg\min_{P} \left\{ \gamma\left( -x_{1} \right) \right\}=arg\min_{P} \{\hat{\delta}\left( x_{1} \right)\}$ $\gamma\left( -x_{1} \right)=N+\hat{\delta}(x_{1})$

Let $j=N \to\hat{\delta}\left( x_{N} \right)=\sum_{i=0}^{N-1} (N-1-i)\cdot p_{i+1}$

$$\gamma\left( x_{N} \right)=p_{1}+{2p}_{2}+\ldots+i\cdot p_{i}+\ldots+\left( N-1 \right)p_{N-1}+\ldots+Np_{N}$$

$$=N\cdot\sum_{i=1}^{N} p_{i}-\sum_{i=0}^{N-1} \left( N-1-i \right)\cdot p_{i+1}=N-\hat{\delta}\left( x_{N} \right)$$

It follows that:

$$arg\max_{P} \{{\gamma(x}_{N})\}=arg\min_{P} \left\{ \gamma\left( -x_{N} \right) \right\}=arg\min_{P} \{\hat{\delta}(x_{N})\}$$

Let $1<j<N$

$$\hat{\delta}\left( x_{j} \right)=\sum_{i=0}^{j-1} \left( j-1-i \right)\cdot p_{i+1}+\sum_{i=j}^{N-1} (i-j+1)\cdot p_{i+1}$$

$$\gamma\left( x_{j} \right)=\sum_{i=0}^{j-1} \left( i+N-j+1 \right)\cdot p_{i+1}+\sum_{i=j}^{N-1} (N-1+j-1)\cdot p_{i+1}$$

$$\sum_{i=0}^{j-1} \left( i+N-j+1 \right)\cdot p_{i+1}=\left( N-j+1 \right)\cdot p_{1}+\left( N-j+2 \right)\cdot p_{2}+\ldots+N\cdot p_{j}$$

$$=N\sum_{i=1}^{j} p_{i}-\sum_{i=0}^{j-1} \left( j-1-i \right){\cdot p}_{i+1}$$

$$\sum_{i=j}^{N-1} \left( N-i+j-1 \right)\cdot p_{i+1}=\left( N-1 \right)\cdot p_{j+1}+\left( N-2 \right)\cdot p_{j+2}+\ldots+\left( j-1 \right)\cdot p_{N-1}+j\cdot p_{N}$$

$$=N\cdot\sum_{i=j+1}^{N} p_{i}-\sum_{i=j}^{N-1} (i-j+1)\cdot p_{i+1}$$

$$\gamma\left( x_{j} \right)=N\cdot\sum_{i=0}^{N} p_{i}-\sum_{i=0}^{j-1} \left( j-1-i \right)\cdot p_{i+1}-\sum_{i=j}^{N-1} (i-j+1)\cdot p_{i+1}$$

$$\boldsymbol{=}N-\sum_{i=0}^{j-1} \left( j-1-i \right)\cdot p_{i+1}-\sum_{i=j}^{N-1} (i-j+1)\cdot p_{i+1}$$

$$\boldsymbol{=}N-\hat{\delta}\left( x_{j} \right)$$

It follows that:

$$arg\max_{P} \left\{ \gamma\left( x_{j} \right) \right\}=arg\min_{P} \left\{ \gamma\left( -x_{j} \right) \right\}=arg\min_{P} \{\hat{\delta}\left( x_{j} \right)\}$$

Where:

$$\gamma\left( -x_{j} \right)=N+\hat{\delta}\left( x_{j} \right)$$

***Q.E.D***

# **BIBLIOGRAPHY**

| [1] | T. O. Kvalseth, "The lambda distribution and its applications to categorical summary measures," *Advances and Applications in Statistics,* vol. 24, no. 2, pp. 83-106, 2011. |
| --- | --- |
| [2] | J. Lin, "Divergence measures based on the Shannon entropy," *IEEE Transactions on Information Theory,* 1991. |
| [3] | E. M. Stein and R. Shakarchi, Fourier Analysis an Introduction p.134, New Jersy: Princeton University Press, 2003. |
| [4] | Herbert.F.Weisberg, Central Tendency and Variability, p35, SAGE Publication, 1992. |
| [5] | T. O. Kvalseth, "Evenness indicies once again: critical analysis of properties," *Springerplus,* p. 4:232, 2015. |
| [6] | F. Gosselin, "Lorenz Partial Order: the Best Known Logical Framework to Define Evenness Indicies," *Community Ecology,* pp. 197-207, 2001. |
| [7] | G. Koshevoy and K. Mosler, "The Lorenz Zonoid of a Multivariate Distribution," *Journal of the American Statistical Association,* vol. 91, no. 434, p. 873, 1996. |
| [8] | R. Bathia and D. Chander, "A better Bound on the variance," *The American Mathematical Monthly,* pp. 353-357, 2000. |
| [9] | G. LV and Y. W.-Y. Xu, "On a new class of measures for health inequality based on ordinal data," *Journal of Economic Inequality,* pp. 465-477, 2015. |
